# Second victim syndrome in surgeons: systematic review and meta-analysis of the impact of adverse events on surgeons

**DOI:** 10.1093/bjs/znaf258

**Published:** 2026-01-07

**Authors:** James Bryan, Adele Ketley, Kate Cavanagh, Carly Bisset, Susan Moug, Lynda Wyld, Jenna Morgan

**Affiliations:** School of Medicine and Population Health, Faculty of Medicine, Dentistry and Health, University of Sheffield, Sheffield, UK; Doncaster and Bassetlaw Teaching Hospitals NHS Foundation Trust, Jasmine Centre, Doncaster, UK; School of Medicine and Population Health, Faculty of Medicine, Dentistry and Health, University of Sheffield, Sheffield, UK; School of Psychology, University of Sussex, Brighton, UK; School of Medicine, Dentistry & Nursing, University of Glasgow, Glasgow, UK; School of Medicine, Dentistry & Nursing, University of Glasgow, Glasgow, UK; Department of General Surgery, Royal Alexandra Hospital, Paisley, UK; School of Medicine and Population Health, Faculty of Medicine, Dentistry and Health, University of Sheffield, Sheffield, UK; Doncaster and Bassetlaw Teaching Hospitals NHS Foundation Trust, Jasmine Centre, Doncaster, UK; School of Medicine and Population Health, Faculty of Medicine, Dentistry and Health, University of Sheffield, Sheffield, UK; Doncaster and Bassetlaw Teaching Hospitals NHS Foundation Trust, Jasmine Centre, Doncaster, UK

## Abstract

**Background:**

Second victim syndrome (SVS) is characterized by negative psychological and psychosomatic effects on a healthcare provider after an adverse care event. The aim of this systematic review and meta-analysis was to characterize the symptoms of SVS experienced by surgeons and factors affecting their impact, as well as understand common coping strategies that surgeons employ to deal with them.

**Methods:**

A systematic review of five electronic databases was conducted without restrictions on publication date or language in January 2025. Second victim syndrome, surgeon, and adverse event and their synonyms were used as search terms. Records were screened, quality assessed, and data extracted by two independent researchers. Both qualitative and quantitative studies were included and narratively synthesized. A meta-analysis was performed using a random effects model to calculate the overall prevalence rates of symptoms and coping methods.

**Results:**

A total of 36 papers were included in the analysis from 6629 retrieved records. Anxiety (56.3% (95% c.i. 45.8% to 66.3%)), guilt (53.8% (95% c.i. 41.3% to 65.8%)), sadness (48.3% (95% c.i. 34.6% to 62.3%)), and sleep disturbance (50.5% (95% c.i. 38.4% to 62.5%)) were the most commonly reported symptoms. Talking to either colleagues (72.5% (95% c.i. 65.6% to 78.4%)) or family/friends (52.0% (95% c.i. 40.6% to 63.2%)) were the most commonly employed coping strategies. The sex and level of experience of the surgeon and the severity of the event were identified as potential predictors of deleterious impact.

**Conclusion:**

SVS significantly impacts surgeons’ global well-being, leading to burnout and attrition. Effective interventions require a multifaceted approach, including peer support, resilience training, and institutional changes that normalize emotional responses, encourage disclosure, and address barriers to seeking help. Targeted support for at-risk groups may also be necessary.

## Teaser

This systematic review and meta-analysis found that adverse events or complications in surgical practice significantly impact the surgeon. Common symptoms are anxiety, guilt, sadness, and sleep disturbance. The currently available research highlights the need for multifaceted interventions, such as peer support and resilience training, to address second victim syndrome and prevent burnout among surgeons.

## Introduction

Adverse events are deviations from a typical care pathway that result in harm or even death to patients during the course of healthcare delivery^1^. Adverse events include errors and complications and these terms are often conflated. An error is a preventable mistake that occurs during an operation or within a course of treatment. It is defined as an unintentional act, by either commission (doing the wrong thing) or omission (failing to do the right thing), that is not considered a known, acceptable risk of the procedure^2^. In contrast, a complication is an unfavourable outcome that is an inherent and known risk of a given procedure, which can occur even when care is delivered to the highest standard^3^. All adverse events can have profound physical, emotional, and psychological consequences for patients; they can undermine trust in the healthcare system and delay recovery^4^. Beyond the immediate impact on health, adverse events may lead to prolonged hospital stays, increased medical costs, and diminished quality of life for the patient and their family^4–6^.

Second victim syndrome (SVS) refers to the psychological and psychosomatic symptoms experienced by healthcare professionals who are involved in adverse patient events or medical errors. The first description of SVS is attributed to Albert Wu in the year 2000^7^ and subsequent studies have shown that up to 59% of physicians in training experience at least one adverse event resulting in SVS symptoms in a preceding year^8^. There is significant heterogeneity in the experience of SVS between individuals, but it can have a profound impact on a healthcare provider’s well-being^9–11^. The literature suggests that the impact of SVS is particularly profound among practitioners in fields such as surgery, anaesthetics, paediatrics, and obstetrics and gynaecology. This is attributed to the nature of the work, the patient population, and the specific challenges inherent in those specialties^12–14^. The terminology of SVS is controversial within the field, particularly as it risks minimizing the patient’s experience and that of their family^15^. Critics also argue that using the word syndrome pathologizes a natural human response, which may contribute to the stigmatization of affected individuals^16^. Although the accepted nomenclature may evolve in the future, SVS is employed here as currently it is the predominant terminology within the relevant literature.

Surgeons deliver care in a way that is different to other healthcare providers. It involves causing harm for therapeutic benefit. The work often involves long hours, complex and time-pressured decision-making, and ongoing professional development of technical and non-technical skills^17^. Surgical training programmes also have high competition ratios when compared with other specialty training programmes^18^. These factors contribute to a strong professional identity and a profound sense of responsibility for patient outcomes^19^. Consequently, when adverse events occur, surgeons may be at an increased risk of developing SVS when compared with other healthcare professionals^20^. The response to adverse events can include psychological effects (for example guilt, shame, anxiety, grief, and depression), cognitive effects (for example burnout, compassion fatigue, and secondary traumatic stress), and social, cultural, spiritual, and physical consequences^21^. The methods of coping with stress differ between individuals and events; Endler and Parker^22^ described three main categories of coping in their Coping Inventory for Stressful Situations. This framework posits three main categories: task-focused, emotion-focused, and avoidance-focused approaches. Strategies aimed at direct problem resolution or impact reduction were considered task-focused approaches. Those focused on managing emotions related to the stressor, including self-preoccupation and anticipatory responses, were classified as emotion-focused approaches. Strategies involving stressor evasion, such as distraction or avoidance of triggering situations, were designated as avoidance-focused approaches. Previous analyses of SVS have found that task-focused strategies are the most commonly employed by healthcare professionals^23^.

Recognizing and addressing SVS is essential for recovery. Scott *et al*.^24^ have outlined six key stages of recovery (identified through interviewing healthcare professionals who have experienced SVS): responding to the initial incident, intrusive self-reflection, rebuilding personal integrity, enduring scrutiny, accessing emotional support, and moving forward. This was built upon by Luu *et al*.^25^, who suggested a simplified timeline of events, with stages entitled: the kick, the fall, the recovery, and the long-term impact. The kick refers to the initial visceral shock of the event characterized by a physiological stress response, which is similar to the first stage in the Scott *et al*.^24^ model. After the initial shock, in the fall, surgeons describe an interval of spiralling out of control, feeling a dark cloud or ‘pall’ over everything. This phase is characterized by intrusive thoughts, searching for answers to determine fault, and worrying about professional reputation, grouping together the second, third, and fourth stages of the Scott *et al*.^24^ model. The recovery, which is equivalent to the fifth and sixth stages of the Scott *et al*.^24^ model, may involve talking to colleagues and reflection. There is an additional stage in the Luu *et al*.^25^ model, which acknowledges the cumulative long-term impact of incidents on surgeons. For some, this is negative; it is an erosion of their sense of self, leading them to change their practice or consider leaving the profession. For others, it leads to personal growth and development.

In both of these models, a supportive workplace culture, including peer and institutional backing, is crucial in fostering recovery for healthcare professionals. In the absence of such support, maladaptive coping strategies may arise, negatively affecting the provider’s mental and physical health and potentially compromising the quality of patient care. However, interventions designed to support affected individuals remain limited, and organizational and cultural barriers often impede progress in this area^26,27^.

The aim of this systematic review was to synthesize the existing evidence on surgeons’ experiences with SVS. It explores the prevalence and impact of SVS, examines the range of responses observed, and identifies factors that may influence these outcomes. It also looks at the support systems and coping strategies that surgeons employ to deal with the impact of SVS. There have been previous reviews of this subject, with regard to both surgeons and the wider healthcare team^21,23,28,29^.

## Methods

This systematic review was registered in PROSPERO, the international prospective register of systematic reviews (registration number 614066, 8 January 2025), and it was conducted according to the PRISMA guidelines^30^.

A literature search was performed in five bibliographic databases: MEDLINE, Scopus, Web of Science, APA PsychInfo, and Cochrane Library. The search strategy used three key elements with synonyms: surgeon (surgeon, surg* trainee, and surg* resident), adverse event (adverse event, adverse clinical event, complication*, and error), and impact (second victim syndrome, burnout, stress, well-being, psychological impact, emotional impact, and compassion fatigue).

This review included primary research studies that examined the physical, psychological, or professional impact (outcome) of adverse clinical events (intervention/exposure) on surgeons of any specialty or training level (population). Studies that detailed or evaluated interventions or support systems for this population were also included. No specific comparison group was required.

The review was limited to primary research (study design); reviews, editorials, and expert opinion pieces were excluded. Additional exclusion criteria included studies not published in English, those not involving surgeons, or those unrelated to adverse clinical events. Where multiple publications reported on the same population, the study with the most participants or the longest follow-up was selected. The screening process for abstracts and then full texts was conducted independently by two researchers (J.B. and A.K.), with any disagreements resolved by the senior author (J.M.), using Rayyan (Rayyan Systems, Cambridge, MA, USA).

Data were extracted by the lead author (J.B.) into Google Sheets (Google, Mountain View, CA, USA). Study design and demographic data for participants were extracted from all included papers. Data were separated into qualitative and quantitative results. Outcomes were categorized into: impact on the surgeon, factors affecting the response, and intervention or coping strategies employed. Coping strategies were classified according to the Endler and Parker^22^ model on coping after stressful events.

Pooled analysis of quantitative data was performed where possible. Due to an expected significant heterogeneity in reporting of outcome measures between papers, a random effects model (DerSimonian–Laird) was used in R version 4.4.2 (R Foundation of Statistical Computing, Vienna, Austria). The overall prevalence for each symptom and coping measure was calculated, as well as the 95% confidence interval and *I*^2^ statistic to assess heterogeneity. Where only percentage data were available, the authors of the paper were contacted to provide the absolute number. If this was not supplied, the absolute number was calculated using the population size and the percentage, and rounded accordingly.

The framework method was used for extraction and analysis of qualitative data^31^. A deductive approach was used primarily, with themes identified from the quantitative papers; additional themes were generated inductively through familiarization with the included qualitative papers. Verbatim quotes were extracted and indexed manually (by the lead author), then charted into a matrix in Google Sheets (Google) where quotes were compared by theme and individual code. Themes were discussed and agreed with the research team before analysis. These data were then summarized into tables including representative quotes, which can be found in the *Supplementary material*. This approach was chosen due to its suitability for large data sets and ability to use both inductive and deductive processes. The charting stage of this process also facilitated comparison of individual quotes within the context of their original paper and allowed comparison with quotes in other papers in which similar themes were identified.

Included papers were quality appraised using the Mixed Methods Appraisal Tool (MMAT)^32^. Quality appraisal was performed by two authors (J.B. and A.K.) independently, with discrepancies discussed and agreed with the senior author (J.M.).

## Results

**Fig. 1:**
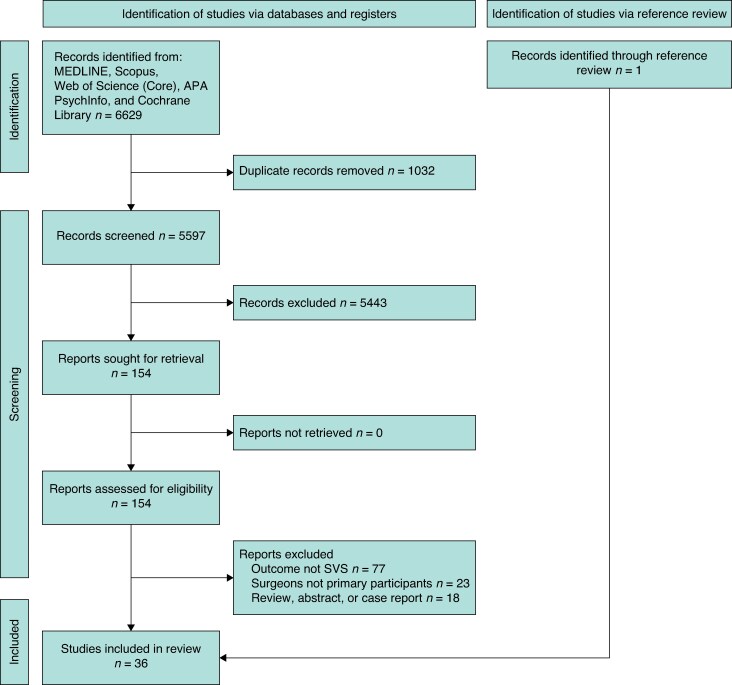
PRISMA flow chart SVS, second victim syndrome.

A total of 6629 records were retrieved from the database search on 8 January 2025, of which 1032 duplicates were excluded. Duplicates were initially identified with a duplicate screening tool in Rayyan (Rayyan Systems); these were then confirmed and removed individually. Abstract, title, and keyword screening was carried out on 5597 unique records, through which a further 5443 records were excluded. Full text analysis was carried out on 154 papers, from which 35 papers were included for final analysis. A single additional paper was identified through reference review of the included articles. Reasons for article exclusion are included in the PRISMA flow chart (*Fig. 1*).

### Quality appraisal of papers

All studies met quality assessment inclusion criteria. A summary of the appropriate sections of the MMAT scores can be found in *Table S1*. Answers to the relevant questions in the tool are: yes, no, and not sure; these are represented in the table by green, red, and yellow boxes respectively.

### Description of articles

The majority of papers reported data from cross-sectional surveys (25 of 36 (69.4%))^33–57^. Nine papers (25.0%) presented only qualitative data from semi-structured interviews^25,58–63^. Two papers (5.6%) presented mixed methods data from a combination of survey and interviews^64,65^. Data from North American and European surgeons made up the majority of papers (12 papers from Europe and 16 papers from North America), with a lower number from Asia (4 papers), Africa (2 papers) and Oceania (2 papers). Twenty-four studies (66.7%) included data from surgical trainees. All surgical specialties were represented in at least one paper. Data collection methods, sample size, and demographics of the surgeons for each study are shown in *Table 1*. The emergent themes of impact on the surgeons were psychological, psychosomatic, professional, and social manifestations. The themes identified concerning support were coping methods and available support, desired support, and barriers affecting engagement.

**Table 1:** Summary of included papers

Study, year	Country	Type	Sample size, *n*	Male	Specialty	Trainees
Akyol *et al*.^33^, 2022	Turkey	Survey	480	422 (87.9)	General surgery 480 (100.0)	0 (0.0)
Al-Ghunaim *et al*.^58^, 2022	UK	Semi-structured interviews	14	11 (78.6)	Neurosurgery 4 (28.6) Urology 6 (42.8) Otorhinolaryngology 2 (14.3) Plastic surgery 1 (7.1) General surgery 1 (7.1)	5 of 14 (35.7)
Balogun *et al*.^59^, 2015	Canada	Semi-structured interviews	23	16 (69.6)	Neurosurgery 12 (52.2) General surgery 8 (34.8) Orthopaedics 1 (0.4) Vascular 1 (0.4) Otorhinolaryngology 1 (0.4)	23 of 23 (100.0)
Balogun *et al*.^60^, 2023	Nigeria	Semi-structured interviews	31	21 (67.7)	Orthopaedics 7 (22.6) General surgery 6 (19.4) Neurosurgery 4 (12.9) Cardiothoracic Surgery 3 (9.7) Ophthalmology 3 (9.7) Urology 2 (6.5) Plastic surgery 2 (6.5) Otorhinolaryngology 2 (6.5) Paediatric surgery 1 (3.2) Gynaecology 1 (3.2)	31 of 31 (100.0)
Bamdad *et al*.^61^, 2023	USA	Semi-structured interviews	28	15 (53.6)	General surgery 28 (100.0)	28 of 28 (100.0)
Berman *et al*.^34^, 2021	USA	Survey	413	281 (68.0)	Paediatric surgery 413 (100.0)	Not specified
Biggs *et al*.^35^, 2020	UK	Survey	82	Not reported	Colorectal surgery 68 (82.9) Hepatobilliary surgery 5 (6.1) Upper gastrointestinal surgery 7 (8.5) Vascular 2 (2.4) Cardiothoracic surgery 2 (2.4)	16 of 82 (19.5)
Chauvet *et al*.^36^, 2023	France	Survey	72	51 of 72 (70.8)	Gynaecology 72 (100.0)	0 of 72 (0.0)
Choi *et al*.^37^, 2024	Canada	Survey	66	Not reported	Vascular surgery 66 (100.0)	14 of 66 (21.2)
Chung *et al*.^38^, 2024	USA	Survey	467	363 of 467 (77.7)	Urology 467 (100.0)	74 of 467 (15.8)
Collings *et al*.^39^, 2025	Australia	Survey	727	296 of 727 (40.7)	Gynaecology 727 (100.0)	107 of 727 (14.7)
D’Angelo *et al*.^40^, 2021	USA	Survey	168	94 of 168 (56.0)	General surgery 168 (100.0)	92 of 168 (54.8)
Drudi *et al*.^41^, 2023	Canada	Survey	65	45 of 65 (69.2)	Vascular surgery 65 (100.0)	0 of 65 (0.0)
Ginzberg *et al*.^65^, 2024	USA	Survey and interviews	93 (survey) 23 (interviews)	49 of 93 (53.3) 13 of 23 (56.5)	Cardiothoracic surgery 6 (6.5) General surgery 37 (39.8) Orthopaedic Surgery 8 (8.6) Otorhinolaryngology 14 (15.1) Plastic surgery 12 (12.9) Urology 10 (10.8) Vascular surgery 6 (6.5)	93 of 93 (100.0)
Han *et al*.^42^, 2017	USA	Survey	126	97 of 126 (77.0)	Cardiac surgery 6 (4.7) General surgery 65 (51.5) Paediatric surgery 12 (9.5) Thoracic surgery 10 (7.9) Transplant surgery 5 (3.9) Trauma surgery 22 (17.4) Vascular surgery 10 (7.9) Other 32 (25.3)	0 of 126 (0.0)
He *et al*.^43^, 2023	China	Survey	1062	802 of 1062 (75.5)	Upper gastrointestinal surgeons who perform radical gastrectomy 1062 (100.0)	0 of 1062 (0.0)
Hsiao and Kopar^64^, 2025	Canada	Survey, focus group, and semi-structured interviews	44 (survey) 7 (focus group and semi-structured interviews)	Frequency not specified	Transplant surgery Colorectal surgery Cardiothoracic surgery General surgery Ophthalmology Frequency not specified	44 of 44 (100.0)
Jain *et al*.^44^, 2022	South Asian Collaborative	Survey	658	490 of 658 (74.5)	General surgery 287 (43.6) Gynaecology 66 (10.0) Orthopaedics 50 (7.6) Plastic surgery 47 (7.1) Paediatric surgery 44 (6.7) Urology 43 (6.5) Gastrointestinal surgery 20 (3) Surgical oncology 19 (2.9) Otorhinolaryngology 16 (2.4) Neurosurgery 15 (2.3) Cardiac surgery 15 (2.3) Breast/endocrine 5 (0.8) Other 31 (4.7)	0 of 658 (0.0)
Khansa *et al*.^45^, 2022	USA	Survey	125	55 of 125 (44.0)	Plastics 53 (42.4) Other 72 (57.6)	125 of 125 (100.0)
Lin *et al*.^46^, 2023	USA	Survey	63	49 of 63 (77.8)	Paediatric surgery 73 (100.0)	10 of 63 (13.7)
Lu *et al*.^62^, 2020	USA	Semi-structured interviews	23	9 of 23 (39.1)	General surgery Surgical oncology Acute care surgery Cardiothoracic surgery Breast surgery Vascular surgery Colorectal surgery Otolaryngology Plastic surgery Urology Frequency not specified	0 of 23 (0.0)
Luu *et al*.^25^, 2012	Canada	Semi-structured interviews	20	15 of 20 (75.0)	General surgery 13 (65.0) Neurosurgery 3 (15.0) Cardiac surgery 1 (5.0) Urology 1 (5.0) Gynaecology 1 (5.0) Vascular surgery 1 (5.0)	8 of 20 (40.0)
McLaren *et al*.^47^, 2021	UK	Survey	36	36 of 36 (100.0)	Otorhinolaryngology 36 (100.0)	36 of 36 (100.0)
O’Meara *et al*.^48^, 2022	Ireland	Survey	16	Frequency not specified	Urology 16 (100.0)	16 of 16 (100.0)
Øyri *et al*.^63^, 2023	Norway	Semi-structured interviews	15	11 of 15 (73.3)	Gastrointestinal surgery 7 (46.7) Cardiothoracic surgery 6 (40.0) General surgery 1 (6.7) Orthopaedics 1 (6.7)	0 of 15 (0.0)
Patel *et al*.^49^, 2010	USA	Survey	123	110 of 123 (89.4)	General surgery 75 (61.0) Trauma 40 (32.5) Critical care 29 (23.6) Vascular surgery 18 (14.6) Orthopaedic surgery 15 (12.2) Breast surgery 13 (11.5) Other surgical specialties <10.0%	0 of 123 (0.0)
Pinto *et al*.^66^, 2013	UK	Semi-structured interviews	27	22 of 27 (81.4)	General and vascular surgery 27 (100.0)	10 of 27 (37.0)
Pinto *et al*.^50^, 2014	UK	Survey	54	32 of 54 (59.3)	General surgery 32 (59.3) Vascular surgery 22 (40.7)	32 of 54 (59.3)
Sandhu *et al*.^51^, 2023	USA	Survey	25	17 of 25 (68.0)	General surgery 17 (68.0) Plastic surgery 7 (28.0) Urology 1 (4.0)	25 of 25 (100.0)
Sikakulya *et al*.^52^, 2024	Uganda and Eastern Democratic Republic of Congo	Survey	198	165 of 198 (83.3)	General surgery 94 (47.5) Gynaecology 58 (29.3) Orthopaedic surgery 32 (16.2) Neurosurgery 2 (1.0) Other 12 (6.1)	115 of 198 (58.1)
Sligter *et al*.^53^, 2020	Netherlands	Survey	292	250 of 292 (85.6)	Orthopaedic surgery 292 (100.0)	61 of 292 (20.1)
Thompson *et al*.^54^, 2017	UK	Survey	167	102 of 167 (64.4)	General surgery 94 (58.0) Trauma and orthopaedics: 24 (15.0) Vascular surgery 10 (6.0) Otolaryngology: 10 (6.0) Urology 7 (4.0) Cardiothoracic surgery 5 (3.0) Plastic surgery 5 (3.0) Neurosurgery 2 (1.0) Oral and maxillofacial surgery 2 (1.0) Paediatric surgery 2 (1.0) Remote and rural surgery 2 (1.0) Other 2 (1.0)	167 of 167 (100.0)
Turner *et al*.^55^, 2022	UK	Survey	445	315 of 445 (70.8)	Academic surgery 20 (4.5) Cardiothoracic surgery 3 (0.7) General surgery 130 (29.2) Neurosurgery 8 (1.8) Oral and maxillofacial surgery 12 (2.7) Ophthalmology 20 (4.5) Otolaryngology 18 (4.0) Paediatric surgery 34 (7.6) Plastic surgery 18 (4.0) Trauma and orthopaedic surgery 72 (16.2) Urology 89 (20.0) Vascular surgery 21 (4.7)	70 of 445 (15.7)
Varughese *et al*.^56^, 2014	Australia and New Zealand	Survey	586	Frequency not specified	Gynaecology 586 (100.0)	96 of 586 (16.4)
Vitous *et al*.^67^, 2022	USA	Semi-structured interviews	46	38 of 46 (82.6)	General 19 (41.3) Colorectal 14 (30.4) Transplant 1 (2.2) Endocrine 2 (4.3) Surgical critical care 8 (17.4) Trauma 4 (8.6) Child thoracic 1 (2.2) Surgical oncology 4 (8.6) Plastic surgery 1 (2.2)	8 of 46 (17.4)
Yaow *et al*.^57^, 2024	Singapore	Survey	196	107 of 196 (54.6)	Breast surgery 9 (4.6) Cardiothoracics 1 (0.5) Colorectal 14 (7.1) Otorhinolaryngology 7 (3.6) General surgery 10 (5.1) Hand surgery 9 (4.6) Head and neck 3 (1.5) Hepatopancreatobiliary surgery 5 (2.6) Maxillofacial 6 (3.1) Neurosurgery (1.5) Gynaecology 25 (12.8) Ophthalmology 4 (2.0) Orthopaedic surgery 35 (17.9) Paediatric surgery 6 (3.1) Plastic surgery 11 (5.6) Surgical oncology 6 (3.1) Trauma 2 (1.0) Upper gastrointestinal surgery 2 (1.0) Urology 23 (11.7) Vascular surgery 5 (2.6) Other 10 (5.1)	63 of 196 (32.0)

Values are *n* (%) or *n* of *n* (%) unless otherwise indicated.

The quantitative data pertaining to ‘impact on the surgeon’ are summarized in *Table 3*.

### Psychological impact

The most common impact of adverse events on surgeons were emotional manifestations of SVS (*Table 2*). Feelings of sadness or low mood were reported by 14 papers with a pooled prevalence of 48.0% (95% c.i. 24.234.6% to 59.762.3%)^33–38,42,44,45,52,53,55,57,65^. In the context specifically of patient mortality, it was reported by one study at 90.6%^33^. When referring to depression, the rate varied between 4.8% and 22.0%^44,45,48,53,55^. However, the only paper that scored this with a validated method (Hospital Anxiety and Depression Scale) found the prevalence to be 4.8% (14 of 292), which, although lower than the other studies, was still higher than the general population, where local normative values of 3.0% are reported^53^. Some surgeons reported that they considered suicide (10 of 658 (1.5%))^44^. Guilt was also a commonly reported symptom with a pooled prevalence of 53.8% (95% c.i. 41.3% to 65.8%)^33,35,36,38,42,44,48,52,57^. A theme that compounded the effect on mood was the sense of isolation. Surgeons mentioned that they believed their reactions were unique, making them feel like an ‘outlier’^25^. When one surgeon heard that their reaction was not unusual they remarked: ‘Good, I’m glad to hear it. It’s lonely’^25^.

**Table 2:** Meta-analysis of the impact of adverse events on surgeons

Category	Symptoms	Pooled frequency	Percentage (95% c.i.)	*I* ^2^ (%)	Number of studies
Emotional	Sadness/low mood	1526 of 3182	48.0 (24.2,59.7)	99.0	14^33,35–38^ ^42^ ^44,45,48,52,53,55^ ^57,65^
	Guilt	1117 of 2286	53.8 (41.3,65.8)	98.7	10^33,35,36,38,42,44,48,57^
	Anxiety	1472 of 2684	56.3 (45.8,66.3)	99.2	10^33,35,37,38,42,43,45,48,57^
	Stress	213 of 676	17.1 (3.3,57.5)	98.9	2^33,57^
	Rumination	994 of 2040	54.5 (32.9,74.2)	99.5	5^33,35,39,44,65^
	Shame/embarrassment	805 of 2901	30.7 (17.8,48.2)	99.2	9^33,35,36,38,39,42,44,57,65^
	Worry for patient/patient’s family	614 of 1335	52.8 (32.6,71.7)	98.1	4^33,35,44,52^
	Fear of litigation/professional consequences	306 of 1319	24.1 (12.5,41.8)	90.7	4^33,37,44,52^
	Anger	354 of 1999	17.2 (10.4,27.1)	89.4	9^33,35,36,38,42,48,52,55,57^
	Disappointment	219 of 562	44.1 (20.6,71.2)	95.8	2^33,35^
	Loneliness	112 of 467	24.0 (–)	–	1^38^
	No negative feelings	32 of 1215	2.9 (1.3,6.3)	46.5	4^33,36,38,57^
Physical/psychosomatic	Sleep disturbance	1795 of 3222	50.5 (38.4,62.5)	99.4	10^33,37–39,44,45,48,55,57,65^
	Loss of appetite	109 of 663	7.7 (13.9,32.4)	96.4	2^38,57^
	Weight gain	14 of 270	5.2 (1.5,16.3)	0.0	2^45,57^
	Weight loss	10 of 270	3.4 (0.7,13.9)	30.1	2^45,57^
	Headache	209 of 1840	11.2 (7.3,16.9)	57.0	5^38,44,45,55,57^
	Gastrointestinal symptoms (nausea, abdominal pain etc.)	328 of 1925	13.6 (5.6,29.7)	95.6	6^38,39,45,48,55,57^
	Cardiovascular/respiratory symptoms (palpitations, shortness of breath etc.)	214 of 941	14.8 (2.9,50.6)	96.6	3^33,48,55^
	Musculoskeletal symptoms (back pain, muscle ache, joint pain etc.)	232 of 727	31.9 (–)	–	1^39^
	Lethargy	3 of 196	1.5 (–)	–	1^57^
	Tremor	16 of 663	1.9 (0.4,7.8)	25.4	2^38,57^
	Psoriasis flare	1 of 196	0.5 (–)	–	1^57^
Professional	Reduced job satisfaction/interest in work	321 of 1456	20.5 (9.3,39.4)	96.10	4^37–39,57^
	Impaired performance at work	25 of 197	12.8 (8.1,19.9)	0.0	2^45,49^
	Low self-esteem/confidence in ability	669 of 2020	35.8 (21.2,54.0)	99.3	7^33,36–39,52,65^
	Urge to leave profession	285 of 1337	19.6 (7.2,43.9)	93.9	3^43,57,65^
Social	Loss of interest in previously enjoyable activities	250 of 480	52.0 (–)	–	1^33^
	Strained relationships with family and friends	270 of 1254	24.5 (9.6,50.6)	95.5	3^35,39,55^

Anxiety was another commonly reported symptom with a pooled prevalence of 56.3% (95% c.i. 45.8% to 66.3%)^33,35,39,42,43,45,48,52,55,57^. Sligter *et al*.^53^ reported the prevalence of anxiety, using the Hospital Anxiety and Depression Scale, to be 8.3%, compared with a general population prevalence of 6%. Intrusive rumination was also reported by a significant proportion of surgeons (54.5% (95% c.i. 32.9% to 74.2%))^33,35,39,44,65^. These feelings were reported by groups in most geographical and cultural areas represented in the research reviewed. This anxiety appears to have two components: the initial ‘kick’—a visceral, physiological response where surgeons report ‘tachycardia and some unease’ and longer-term anxiety related to self-confidence, reputational damage, and worry for the patient^25,59,62,65^. Surgeons reported these feelings being pervasive outside of work, affecting both their sleep and their ability to engage with other activities^62,65^. One surgeon recounted: ‘I had a hard time sleeping for a while after a complication. Sometimes I would feel my heart racing during the day or it would be difficult to breathe’^62^.

Feelings of shame and embarrassment were also commonly reported (30.7% (95% c.i. 17.8% to 48.2%))^33,35,39,42,44,57,65^. A resident surgeon expressed such feelings, wondering if their error was ‘unforgivable and is it going to affect people’s professional opinion of me’^66^. Along with fear of professional restriction or legal action (24.1% (95% c.i. 12.5% to 41.8%))^33,37,44,52^, worry for the well-being of the patient and their family was commonly reported (52.1% (95% c.i. 32.6% to 71.7%)), with surgeons describing a feeling of failure in the context of ‘having someone trust you to do a major surgery and then having [a complication] something like that happen’^33,35,44,52^. Anger made up a smaller percentage of emotional impact (17.2% (95% c.i. 10.4% to 27.1%))^35,36,38,42,48,52^  ^55,57^. This anger was often directed at themselves with one surgeon saying: ‘I do it just to punish myself, just to torture myself, just to flagellate myself. I go over and over and I beat myself up. And I tell myself I’m not worthy’^25^.

Chung *et al*.^38^ found that, for the majority of surgeons, the emotional impact lasted <6 months (345 of 467 (74%)), but a significant proportion still had ongoing symptoms after 1 year (61 of 467 (13%)). This was echoed by Khansa et al.^45^, who found that 12.2% (9 of 74) had emotional sequelae for >1 year^45^. One surgeon described this experience as one that ‘certainly haunted me for a very long time, especially [since] I was new to the institution. I was embarrassed. There was many levels of guilt and I kind of thought that people will lose confidence in my abilities’^65^. There was a small subgroup of surgeons described in some cohorts who denied any emotional response at all (2.6% (95% c.i. 1.3% to 6.3%))^33,36,38,57^. This was expressed as: ‘Any error I have made in the operating room has minimal consequences for me’^63^.

The symptoms described by some surgeons experiencing psychological impacts of adverse events in their patients has significant crossover with acute stress disorder or post-traumatic stress disorder (PTSD). These surgeons described re-experiencing symptoms, hyperarousal, avoidance behaviour, and emotional numbing^33–37,39,44,65^. Hyperarousal manifested as difficulty sleeping and physical symptoms of anxiety (such as palpitations); others described vigilance behaviour, for example constantly checking for updates about the patient even whilst not at work^25,62,65^. One surgeon described this inability to switch off from work, constantly ‘Checking my phone… I’m trying to help my kids with homework and I’m thinking about my patient’^65^. Avoidance-type responses described by surgeons were making changes to professional practice like taking less risk, with one stating it might make them ‘much less prone to taking any form of risk… and sometimes that’s not necessarily in the best interests of the patient’, or changing the scope of the operations they perform, as well as leaving the profession entirely^25,65^. Re-experiencing symptoms is described both immediately after the event and for years afterwards, especially around the anniversary of the event, with one surgeon recalling a patient death by saying: ‘I think of her around every Easter’^65^. Finally, some surgeons described developing emotional numbing, with one resident fearing they might ‘stop caring, just become desensitized to it, which also isn’t good’^61^.

Some studies used validated tools to screen for clinical PTSD and traumatic stress of clinical concern^36,41,48,50,53,54^. Two studies used the Impact of Event Scale (IES), which is a validated 15-item tool, where participants score how frequently they experience intrusive and avoidant symptoms^41,50^. Two other studies used a revised 22-item tool (Impact of Event Scale-Revised (IES-R)) with different thresholds for stress disorders^36,54^. Two studies used the Primary Care PTSD screening tool (PC-PTSD-V)^48,55^. Sligter *et al*.^53^ used the Trauma Screening Questionnaire (TSQ). The TSQ is a 10-item screening tool with binary responses; a score of ≥6 suggests a provisional diagnosis of PTSD.

The prevalence of PTSD after an adverse event ranged between 0.3% and 36.2%^36,41,48,50,53–55^. In a study of 47 general and vascular surgeons, 17 (36.2%) scored above the IES cut-off point of 19, which indicates traumatic stress of clinical concern^50^. Drudi *et al*.^41^ found that 20 of 65 participants had an IES score >24, where PTSD can be considered as a diagnosis. In a study of 167 UK surgical trainees, 13.7% of participants (23 of 167) had an IES-R score of ≥33, which is indicative of acute stress disorder or PTSD^54^. Acute stress disorder, indicated by symptoms lasting <1 month, was observed in 3.6% (6 of 167), whereas 17 of 167 (9.6%) had symptoms lasting >1 month (PTSD)^54^. Chauvet *et al*.^36^ found that 11.5% (6 of 52) had an IES-R score of ≥36, indicating acute stress disorder or PTSD. O’Meara *et al*.^48^ used PC-PTSD-V, which showed that 1 of 16 respondents (6.25%) met the criteria for PTSD. Sligter *et al*.^53^ used the TSQ and only 1 of 292 respondents (0.3%) screened positive for PTSD using this tool.

### Psychosomatic impact

Psychosomatic symptoms were less commonly reported in SVS; displayed in *Table 2*. The most commonly reported manifestation was a disturbance to sleep (50.5% (95% c.i. 38.4% to 62.5%))^33,37,39,44,45,48,49,55,57,65^. This was often reported as insomnia; however, Collings *et al*.^39^ demonstrated that the majority having sleep disturbance experienced frequent waking or interrupted sleep (414 of 727 (56.9%)) and that a further 25.8% (167 of 727) found it difficult to get back to sleep after waking. The primary reason for this disruption to sleep was attributed to intrusive thoughts, which was described as: ‘It’s one of those things where you wake up in the middle of night, you’re like, checklist. Could I have done this? Could I have done this? Could I have done this?’^25,62,65^. Some noted that this sleep disturbance increased the likelihood of further errors^58,60^. Other common symptoms included headache (11.2% (95% c.i. 7.3% to 16.9%)), weight gain (5.2% (95% c.i. 1.5% to 16.3%)), nausea (13.6% (95% c.i. 5.6% to 29.7%)), and palpitations (14.9% (95% c.i. 2.9% to 50.6%))^33,38,39,44^  ^45,48,55,57^.

The duration of symptoms was variable between individuals and between studies, although physical symptoms tended to be shorter-lived than emotional symptoms, with the majority of surgeons experiencing these symptoms for <1 month^21,38,45,54^.

### Professional impact

The most commonly reported professional impact was reduced self-esteem or confidence in one’s ability at work (35.8% (95% c.i. 21.2% to 54.0%))^33,36,37,39,49,52,65^. This crisis of confidence was articulated by a surgeon who felt: ‘It’s like I failed… I’m not entitled to wear my lab coat and my scrubs and be a surgeon… You just feel personally devalued’^25^. This sometimes manifested in coping strategies and making changes to their professional practice, such as becoming more cautious in the cases they operated on, a change in surgical technique, ordering more diagnostic tests, or having a lower threshold for calling a colleague to help^25,33,35,38,53^. Another relatively common professional impact was having decreased job satisfaction (20.5% (95% c.i. 9.3% to 39.4%))^37–39,57,58^. Some surgeons had the urge to leave the profession or retire (19.6% (95% c.i. 7.2% to 43.9%))^38,43,57,58,62,65^. Some regretted joining the profession altogether, saying: ‘In all honesty, I would not have gone into this field if I had to do it again’^65^. Impaired performance or decision-making was also noted by some studies in 12.8% (95% c.i. 8.1% to 19.9%) of respondents^45,49^. A short-term example of this was one surgeon who felt they could not complete the final part of a procedure after a complication, saying: ‘I could have sewn it in myself but by that point I was fairly destroyed’^25^.

### Social impact

The impact of adverse events on surgeons sometimes led to strained relationships with family and friends, as well as colleagues (21.5% (95% c.i. 19.3% to 24%))^35,39,55,65^. One study reported surgeons losing interest in previously enjoyable activities such as hobbies or interests outside of work (250 of 480 (52%)), although this probably has significant crossover with mood disorders^33^.

### Coping strategies

Some coping strategies had distinct crossover between the categories outlined by Endler and Parker^22^ and these instances were included in both groups (*Table 3*). The qualitative papers explored the mindset of surgeons, as well as the specific actions that surgeons had taken to cope; these did not always fit into the Endler and Parker^22^ framework. The common internal coping themes identified were inevitability and contextualization^25,60,61,63,65^. The theme of contextualization is illustrated well by the following quote, where a surgeon balanced negative feelings from a negative outcome with previous positive feelings from positive outcomes: ‘I actually have saved some files of screenshots of very nice reviews that patients—as much as I hate that I’m being rated like a restaurant. I have saved some of them that are meaningful and kind. I will reread those to try and find a perspective’^65^. Some surgeons perceived themselves as being innately more emotionally resilient^25,61^. An example of this is given by a surgeon after experiencing an adverse event: ‘I don’t let it hold me down because I can’t let it distract with the next decision that I have to make 10 min later. This is when my wife tells me that I have no emotions because I have to keep moving forward’^67^.

**Table 3:** Meta-analysis of coping strategies

Coping strategy	Type	Pooled frequency	Percentage (95% c.i.)	*I* ^2^ (%)	Number of studies
Speaking to colleagues or senior surgeon	T/E	2313 of 3283	72.5 (65.6,78.4)	93.9	16^35–37,39–42,44–46,48,49,53,55,57^
Speaking to family or friends	E	1422 of 2824	52.0 (40.6,63.2)	97.9	13^34,37,39,40,42,45,46,48,49,53,55,57^
Exercise	A	919 of 1925	45.3 (31.1,60.4)	98.5	7^34,37,39,40,46,53,57^
Reflection/positive reframing	T/E	195 of 535	43.5 (25.5,64.0)	94.9	6^35–37^ ^41,45,57^
Finding a solution/treating the complication	T	181 of 607	40.8 (15.8,72.7)	98.0	4^35,40,41^ ^53^
Letting time pass	A	95 of 343	39.6 (8.6,83.6)	97.9	3^35,41,57^
Seeking distraction	A	130 of 439	38.0 (16.3,66.3)	97.8	3^35,41,53^
Speaking to patient/patient’s family	T/E	496 of 1656	37.0 (18.7,60.6)	98.4	8^35,40,44–46,49,53,57^
Self-blame/criticizing oneself	E	150 of 789	25.5 (10.5,51.6)	87.9	3^37,41,44^
Making light of the situation	E	15 of 65	23.1 (–)	–	1^41^
Avoidance of certain procedures, situations, or patients	A	167 of 1288	21.5 (7.8,47.2)	97.6	5^34,36,37,44,65^
Internalization/suppression of feelings	E	209 of 1414	16.9 (9.3,28.9)	88.0	6^35,44,46,49,53,57^
Formal counselling/professional help	E	211 of 2393	11.3 (4.8,24.4)	95.4	9^34,42,43,45,46,49,53^ ^57^
Taking action to affect systemic changes	T	26 of 242	11.1 (3.8,28.9)	83.1	2^40,45^
Blaming external factors	E	34 of 315	10.8 (7.5,15.6)	87.3	3^35,40,41^
Contact lawyer or medical defence organization	T	7 of 66	10.6 (–)	–	1^37^
Religion/prayer	E	55 of 1095	7.4 (2.3,21.2)	84.7	6^34,37,41,46,53,57^
Alcohol or other drugs	A	133 of 1998	7.1 (4.4,11.3)	72.1	8^34,35,37,40,44,49,53,57^
Taking time off	A	46 of 1041	4.2 (2.3,7.5)	1.2	5^34,37,45,53,57^
Review of literature or guidelines	T	18 of 554	4.2 (1.3,12.3)	47.6	3^37,53,57^
Hobbies	A	5 of 196	2.6 (–)	–	1^57^
Speaking to a regional or national support service	E	13 of 584	2.0 (0.4,7.8)	16.0	3^48,49,55^
Meditation	E/A	3 of 196	1.5 (–)	–	1^57^

T, task-focused strategy; E, emotion-focused strategy; A, avoidance-focused strategy.

Examples of task-based strategies used by surgeons were: focusing on managing consequences of the adverse event, reviewing literature and guidance on similar cases, and being more vigilant. Varughese *et al*.^56^ also identified the use of quality assurance and key performance indicators as an effective tool for a surgeon to understand their complication rate and compare it with those of their peers and an accepted standard.

The most common coping strategies were talking about the adverse event to a colleague (72.5% (95% c.i. 65.6% to 78.4%))^35–37,39–42,44–46,48,49,53,55,57,64^ or a member of family (52.0% (95% c.i. 40.6% to 63.2%))^34,37,39,40,42,45^  ^46,48,49,53,55,57^. It was not always clear from these papers whether this was focused on the task or the emotion of the event. Quotes from the qualitative papers suggest it is often both. Surgeons described talking to both peer colleagues and more senior mentors. The quotes mentioned the importance of having another surgeon to talk to who both understands the technical aspects of the adverse event and has the ability to empathize with the feelings in the situation^60,61^. An illustration of this sentiment is apparent in this quote: ‘it’s sort of hard to explain to people, when unless you’ve gone through it, you can’t understand’^58^. Another surgeon remarked how talking to other surgeons helped combat the feeling of isolation by saying they wanted to: ‘Talk to people who can relate to what you’re going through and say I’ve, that’s happened to me too, right. So then you don’t feel alone that you’re the only person that messed up’^61^. The next most common strategy was physical exercise (45.3% (95% c.i. 31.1% to 60.4%)), which can be viewed as an avoidance strategy^34,37^  ^39,40,46,53,57^. Other examples of avoidance strategies include participating in hobbies; one surgeon said they coped by: ‘either picking up my guitar or going out for a really nice meal, having just one drink and sitting down and enjoying that meal. You know, just something to kind of divert energy’.

Examples of other task-based strategies were reviewing current literature around the adverse event (3.3% (95% c.i. 1.3% to 12.3%)) or making plans to deal with the problem (29.8% (95% c.i. 15.8% to 72.7%)), as well as taking steps to affect systemic or process changes (11.1% (95% c.i. 3.8% to 28.9%))^35,37,40,41,45,53,57^. This was described by a resident as a way to create meaning: ‘Every major complication I’ve had in residency has in some way changed my practice… I think that like kind of the process they go through to like deal with complications’. Aside from talking about the adverse event, other emotion-based coping strategies included making light of the situation (23.1% (16 of 65)), suppressing negative feelings (16.9% (95% c.i. 9.3% to 28.9%)), or blaming external factors (10.8% (95% c.i. 7.5% to 15.6%))^35,40,41,44,46,49,53^  ^57^. A common theme in the qualitative literature was that complications were easier to view as an inevitable consequence of operating than errors^62,65,66^. Maladaptive avoidance coping strategies were also employed such as an increase in substance use, either drugs or alcohol, in a small proportion of surgeons experiencing SVS (7.1% (95% c.i. 4.4% to 11.3%))^34,35,37,44^  ^50,53,55^.

### Intraoperative coping strategies

A single paper surveyed surgeons on intraoperative coping strategies^40^. The most common strategies used were stopping and taking time to think (55.3% (93 of 168)) and focusing on calming emotions (48.8% (82 of 168)). Other strategies included calling for another surgeon to help, checking to reassess judgement, and making ergonomic adjustments.

### Suggested support and barriers affecting engagement

Surgeons who have experienced SVS expressed a diverse range of needs and desires in terms of support. Many expressed a strong preference for peer support groups, where they can connect with colleagues who understand and can empathize with their experience^33,38,44^. Trainees and less experienced surgeons tended to want mentoring and support from senior surgeons^33,38^. This was described in one account as a ‘one-on-one M&M [Morbidity and Mortality meeting]’, calling the opportunity to debrief with a senior surgeon who could share their own experiences ‘very therapeutic’^65^. Berman *et al*.^34^ acknowledged that some surgeons may require additional training to deliver this support. In addition, many surgeons would like access to educational programmes and training resources that specifically address the psychological impact of adverse events and provide practical coping strategies^33,38,65^. This was summarized as: ‘When you are a medical professional and you’re putting yourself in harm’s way emotionally, you need to be taught how to deal with that’^65^. Many studies suggested that these programmes should be integrated into surgical training and continued professional development^64^  ^33,37,51,55^. Many surgeons expressed that surgical training had not adequately prepared them for the impact of adverse events in their patients when moving into more independent practice^37,47,48,55,63^. A quote from a participant in the study by Choi *et al*.^37^ illustrates this well: ‘We should learn to deal with adverse events in residency or have a system in place to assist trainees and those transitioning into practice’.

Beyond structured programmes, surgeons suggested the need for a workplace culture that fosters open communication and destigmatizes seeking help after adverse events^34–36,38,57^. They suggested incorporating discussions on the emotional impact of adverse events into existing platforms such as Morbidity and Mortality (M&M) meetings. One surgeon described how these meetings currently fail in this regard: ‘everybody in that room is very defensive and aggressively pursues an angle that puts them in the best possible light and professional rivalries exist …I don’t find them cathartic forums for saying that was just terrible wasn’t it’^66^. These discussions should address the psychological impact of adverse events alongside technical aspects, creating a safe space for surgeons to express their emotions and concerns^35,40,57,59,63,65^. Another surgeon noted that ‘the obsession in M&M is, how could you have prevented it, rather than… how is the team handling that?’^65^. Some find that traditional M&M meetings can be accusatory and hostile, which may hinder open discussion, support, and learning^39,42,60^. In the two studies that surveyed surgeons with regard to their satisfaction with the support of their institution after an adverse event, most found the support to be inadequate^34,35^.

Additionally, some surgeons report facing challenges in accessing support due to time constraints or awareness, skepticism with regard to its efficacy, fear about stigma, and unfamiliarity with colleagues^25,63,65,66^. A major barrier is a culture that equates emotional vulnerability with weakness. As one surgeon explained: ‘The moment you show that you’re maybe a little bit weak, that’s bad, right. Surgeons can’t show that they’re weak’^25^. Addressing these barriers is crucial to ensure that surgeons feel comfortable seeking help when needed^34,37,65^.

### Factors affecting response

Several factors can influence the intensity and nature of a surgeon’s response to an adverse clinical event. These factors include the surgeon’s sex and seniority, the severity of the event, and whether the surgeon perceives the event as being contributed to by an error on their part^36,38–41,50,54,55,62^.

### Sex

Sex has been identified as a factor affecting the response to adverse events in several studies. Multiple studies have reported that female surgeons were more likely to report that their physical and mental health were affected when an adverse event occurred^39,52,53,65^. They may be more likely to blame themselves and less likely to see the complication as ‘expected’ or due to external factors^39,52^. Female surgeons more commonly experienced an acute stress reaction (defined as an IES score >24)—11 of 20 (55%) female surgeons compared with 9 of 45 (20%) male surgeons who were surveyed^41^. Differences between the sexes were observed with regard to the use of specific intraoperative coping strategies. Female surgeons were more likely to report ‘focusing on calming themselves down to reduce their own stress response’ (60.1% (45 of 74) *versus* 38.3% (36 of 94)), whereas male surgeons were more likely to report ‘making ergonomic adjustments’ (18.1% (17 of 94) *versus* 2.7% (2 of 74))^40^.

However, other studies have found no relationship between sex and the impact of adverse events^36^  ^44,54,57^. Conversely, Lu *et al*.^62^ found that male surgeons were more likely to report adverse events contributing to burnout than their female colleagues. Male surgeons were more likely to disclose their error to the patient or their family and were more likely to be comfortable talking to a colleague about the adverse event^39,40^.

### Years of experience

Several studies have identified age or years of experience as a factor affecting the response to adverse events^39,52,55^. Collings *et al*.^39^ reported that a significantly higher proportion of obstetricians and gynaecologists with <15 years of experience or current trainees (36 of 357 (10.1%)) had mental health impacts after an adverse event when compared with those with >15 years of experience (12 of 316 (3.8%)). In contrast, Choi *et al*.^37^ have reported significantly higher general distress in attending surgeons (64.7% (33 of 51)) than trainees (33.3% (5 of 15)) after an adverse event, although other symptoms such as sleep disturbance and anxiety remained comparable between the two groups^37^. Trainees were more likely than consultants to have considered leaving the profession due to an adverse event (35.9% (28 of 79))^65^.

Consultant surgeons were more likely to take action for the patient affected and disclose the error/adverse event to the patient or their family than trainees^40^. Consultants were more likely to have developed coping mechanisms and support networks over time^39,40,50,55^. They may also be more likely to view adverse events as learning opportunities, as they have a broader perspective on their careers^39,55,65^.

Some studies showed no difference in emotional and behavioural responses or coping strategies between independent surgeons and trainees^33,37,42^. Berman *et al*.^34^ found that there were no differences in the likelihood of being satisfied with the institutional response to an adverse event according to surgeon age.

### Type of adverse event

The type and severity of an adverse event has been identified as a factor affecting the response in several studies^38,39,41,52,54,55^. Collings *et al*.^39^ identified that adverse events caused the most stress when they resulted in poor patient outcomes or were a result of surgeon error. Similarly, other papers found that, when the adverse event was perceived as an error, the surgeon was more likely to experience sleep problems, anxiety, increased alcohol consumption, and develop PTSD than those experiencing a recognized complication^38,39,41,52,55^. Thompson *et al*.^54^ found that surgeons who had witnessed severe pain, traumatic injury, or massive intraoperative haemorrhage were more likely to experience clinically significant PTSD. In the context of patient mortality, Akyol *et al*.^33^ reported that more surgeons found the death of a younger patient to have a greater emotional impact on them than the death of an older patient (286 of 480 (59.7%)).

However, other studies have found no relationship between the type of adverse event and the impact of adverse events. Whilst this was a hypothesis of the study by Pinto *et al*.^50^, they found no association between the controllability of the cause of adverse event and the severity of the impact on the surgeon. Similarly, two other studies found no association between the type of adverse event and the severity of emotional impact^54,57^.

## Discussion

This systematic review synthesizes evidence from 36 studies with both quantitative and qualitative methodologies, confirming that SVS is a significant occupational risk for surgeons and surgical trainees. The findings demonstrate that adverse patient events can affect many facets of surgeons’ lives. They affect emotions, physical health, professional behaviour, and relationships at work and at home. Common symptoms of low mood, guilt, anxiety, rumination, and sleep disturbance were consistent with previous reviews of surgeons and other healthcare professionals^21,28^. The burden of adverse events may be contributing to the significantly higher rates of anxiety and depression (20% and 24% respectively) observed in surgeons when compared with the general population^68^. The symptoms experienced are often short-lived; however, there seems to be a significant proportion of surgeons who go on to experience long-term or profound effects on their quality of life. Post-traumatic stress-type reactions are relatively common, with a prevalence of between 0.3% and 36.2%. These factors may contribute to burnout, attrition in training, and surgeons leaving the profession^38,43,58,62^. The influence of personal and event factors on the duration and severity of the effect is not yet fully understood. However, it does appear that the sex and level of experience of the surgeon and event severity, as well as the perception of the event as an error, all exert an influence^55^.

Surgeons coped with the impact of adverse events in many different ways and there was no one strategy or strategy type that seemed to work for all. In reality, surgeons used a combination of task-, emotion-, and avoidance-focused strategies. Seeking peer and mentor support was the most commonly employed coping strategy^44,45,55,57,61,69^; this may be because it can be both a task-focused strategy and an emotion-focused strategy that can be tailored to the individual situation. Conversations with colleagues and mentors provide reassurance and validation, alleviating intense emotions by fostering a sense of shared experience, as well as giving practical, task-focused ways to address the practical aspects of an adverse event. Preliminary findings indicate that peer support initiatives are well received, with many participants reporting positive impacts on departmental safety and support culture^42,70^.

Risk factor specific support strategies may also be necessary, as this research suggests female surgeons may respond differently to adverse events compared with their male counterparts. Female surgeons, along with less experienced surgeons, are at a higher risk of experiencing longer-term SVS and may perceive the profession as overwhelming and insufficiently rewarding^39–41,52,53,55^. Personality is known to influence how comfortable a surgeon is with risk and affects decision-making behaviour^71–73^; however, more research is needed to understand the effects of personality type on SVS^17^.

Many surgical trainees found that surgical training did not adequately prepare them for the impact of adverse clinical events; as such, training and support should be integrated into postgraduate surgical curricula^47,48,55,65^. Dealing with the impact of adverse events, along with other non-technical skills, is part of a ‘hidden curriculum’ surgical trainees are expected to pick up through their training^19,73,74^. The transition to independent practice appears to be the time interval during which surgeons are most vulnerable to SVS^37,39^. Support could be delivered to this group before events occur. Resilience training has been shown to be effective in managing stressful situations and may be effective in providing surgeons with tools to deal with an acute stress reaction to an adverse event^75,76^. However, empirical evidence on the long-term effectiveness of such programmes remains limited in this context. The key to an effective targeted intervention may involve trying to identify predictors of more significant impact on the surgeon, as well as identifying peritraumatic factors such as dissociative symptoms that correlate with more severe symptoms^77,78^.

Beyond individual factors, the professional culture plays a significant role in shaping the second victim experience. The culture in surgery is commonly characterized by expectations of perfectionism, infallibility, and emotional stoicism^61,66,67,79^. Internalization as a coping strategy may worsen and prolong symptoms for some^50,51,61^. Surgeons may also have a poor awareness of their own level of emotional stress or psychological difficulties and be less likely to engage in self-initiated support methods^80^. Therefore, externally initiated measures may be necessary in some circumstances, provided these are non-punitive. On the organizational side, formal counselling services, both local and national, are often underutilized^48,49,55,61^. Surgeons express reservations about these services, citing unfamiliarity with support staff and doubts about their effectiveness, as well as trepidation about non-self-initiated measures. Reflective practice can be an effective coping strategy; however, there are still reservations amongst doctors about documenting honest reflections of errors or adverse events in the wake of the Dr Bawa-Garba case^81^. This also extends to reluctance to discuss these circumstances for fear of reputation damage and punitive action^61,65^. Additional barriers to institutional support include insufficient training, unsupportive workplace cultures, and medicolegal fears^25,62,65^. The organizational interventions tend to prioritize technical aspects over emotional consequences, which may further exacerbate these challenges. Such cultural barriers discourage disclosure and hinder recovery, contributing to a cycle where emotional distress and medical errors may perpetuate one another^20,82,83^.

Addressing the emotional aspects of adverse events is critical for breaking this cycle. Initiatives to normalize emotional responses, encourage disclosure, and integrate resilience training into surgical education could help reduce the stigma surrounding SVS. Incorporating tools such as self-assessment resources, confidential support links, and reframing platforms like M&M meetings could provide additional avenues for support. However, integrating SVS-related training into already demanding surgical curricula poses practical challenges. Efforts must also address factors such as the lack of awareness about SVS, the blame culture, reluctance to seek help, and concerns about confidentiality. Organizational leaders should play a pivotal role in fostering a supportive work environment and setting the tone for cultural transformation within the surgical field.

The main strength of this work is the breadth of the studies included, allowing both meta-analysis of quantitative data and integration with qualitative literature. The qualitative component provides useful context and insight into the nuance of the experience, whilst not being able to provide generalizable results alone. This review focuses on surgeons who are a unique group in healthcare provision and identifies subtle differences in the experience of this group when compared with the broader healthcare community.

Significant heterogeneity in the reporting of symptoms and coping strategies limits the ability to generalize the findings. Terms like ‘sadness,’ ‘depression,’ and ‘low mood’ lie on a spectrum of negative affect and functional impact; their inconsistent usage across studies hinders comparisons. Future research would benefit from the adoption of standardized, validated scoring systems to more accurately characterize the severity and nature of surgeons’ emotional responses to adverse events. Similarly, the variability in tools defining and assessing PTSD necessitates a more uniform approach to ensure consistency and comparability.

The MMAT tool was used to assess risk of bias in this study. All of the included studies had clear research aims and appropriate methodology. However, all of the qualitative studies used convenience sampling methods, which subject the findings to selection bias, as surgeons who feel strongly about the subject or are significantly impacted may be more likely to participate. The same bias is true for the cross-sectional surveys, which were voluntary. The response rate varied greatly between studies (10.3–98%), although most achieved a rate >30%.

In addition, the retrospective nature of many of the included studies introduces the potential for recall bias. Although it was not always specified, the length of time between an adverse event and collecting the data on the impact was often different between individuals, even within the same study, which could affect the context reported by individuals. The personal context surrounding the surgeon at the time of the traumatic event, as well as the immediate effect on the surgeon afterwards, should be examined in future research.

Finally, the assessment of adverse event severity and its correlation with second victim experiences presented methodological challenges. Whilst the Clavien–Dindo classification was utilized in some studies, it did not consistently predict the severity of emotional responses. Future studies should strive to develop methodologies that can more accurately capture and adjust for contextual adverse event severity, allowing for a more precise understanding of the relationship between severity and the surgeon’s emotional response.

## Supplementary Material

znaf258_Supplementary_Data

## Data Availability

The authors confirm that the data supporting the findings of this study are available within the article and its *Supplementary material*.

## References

[1] Institute of Medicine . To Err is Human: Building a Safer Health System. Washington, DC: National Academies Press, 2000 25077248

[2] Santos G, Jones MW. Prevention of Surgical Errors. Treasure Island, FL: StatPearls Publishing, 2025 37276278

[3] Giddins G . Surgical complications: errors and adverse events. J Hand Surg Eur 2024;49:142–148 10.1177/17531934231206317 38315132

[4] van der Velden PG, Contino C, Akkermans AJ, Das M. Victims of medical errors and the problems they face: a prospective comparative study among the Dutch population. Eur J Public Health 2020;30:1062–1066 33313817 10.1093/eurpub/ckaa106 PMC7733042

[5] Vincent C, Neale G, Woloshynowych M. Adverse events in British hospitals: preliminary retrospective record review. BMJ 2001;322:517–519 11230064 10.1136/bmj.322.7285.517 PMC26554

[6] Sari ABA, Sheldon TA, Cracknell A, Turnbull A, Dobson Y, Grant C et al Extent, nature and consequences of adverse events: results of a retrospective casenote review in a large NHS hospital. BMJ Qual Saf 2007;16:434–439 10.1136/qshc.2006.021154 PMC2653177 18055887

[7] Wu AW . Medical error: the second victim. BMJ 2000;320:726–727 10720336 10.1136/bmj.320.7237.726 PMC1117748

[8] Strametz R, Koch P, Vogelgesang A, Burbridge A, Rösner H, Abloescher M et al Prevalence of second victims, risk factors and support strategies among young German physicians in internal medicine (SeViD-I survey). J Occup Med Toxicol 2021;16:11 33781278 10.1186/s12995-021-00300-8 PMC8005860

[9] Seys D, Scott S, Wu A, Van Gerven E, Vleugels A, Euwema M et al Supporting involved health care professionals (second victims) following an adverse health event: a literature review. Int J Nurs Stud 2013;50:678–687 22841561 10.1016/j.ijnurstu.2012.07.006

[10] Van Gerven E, Bruyneel L, Panella M, Euwema M, Sermeus W, Vanhaecht K. Psychological impact and recovery after involvement in a patient safety incident: a repeated measures analysis. BMJ Open 2016;6:e011403 10.1136/bmjopen-2016-011403 PMC5013512 27580830

[11] Liukka M, Steven A, Vizcaya Moreno MF, Sara-aho AM, Khakurel J, Pearson P et al Action after adverse events in healthcare: an integrative literature review. Int J Environ Res Public Health 2020;17:4717 32630041 10.3390/ijerph17134717 PMC7369881

[12] Wuthnow J, Elwell S, Quillen JM, Ciancaglione N. Implementing an ED critical incident stress management team. J Emerg Nurs 2016;42:474–480 27236821 10.1016/j.jen.2016.04.008

[13] Boyle D, O’Connell D, Platt FW, Albert RK. Disclosing errors and adverse events in the intensive care unit. Crit Care Med 2006;34:1532–1537 16540948 10.1097/01.CCM.0000215109.91452.A3

[14] Torbenson VE, Riggan KA, Weaver AL, Long ME, Finney RE, Allyse MA et al Second victim experience among OBGYN trainees: what is their desired form of support? South Med J 2021;114:218–222 33787935 10.14423/SMJ.0000000000001237 PMC8018514

[15] Clarkson MD, Haskell H, Hemmelgarn C, Skolnik PJ. Abandon the term “second victim”. BMJ 2019;364:l1233 30917966 10.1136/bmj.l1233

[16] Wu AW, Shapiro J, Harrison R, Scott SD, Connors C, Kenney L et al The impact of adverse events on clinicians: what’s in a name? J Patient Saf 2020;16:65–72 29112025 10.1097/PTS.0000000000000256

[17] Boghdady El, Esmaeili M, Zargaran A, Brennan A, P. Culture in surgery. Bull R Coll Surg Engl 2024;106:38–41

[18] Mellor K, Robinson DB, Luton O, James OP, Powell Agmt, Hopkins L et al Prognostic significance of competition ratios in surgical specialty training selection. Postgrad Med J 1163;98:700–704 10.1136/postgradmedj-2020-139491 37062983

[19] Rivard SJ, Vitous CA, Roo De, Bamdad AC, Jafri MC, Byrnes SM et al “The captain of the ship.” A qualitative investigation of surgeon identity formation. Am J Surg 2022;224:284–291 35168761 10.1016/j.amjsurg.2022.01.010 PMC9531326

[20] Shanafelt TD, Balch CM, Bechamps G, Russell T, Dyrbye L, Satele D et al Burnout and medical errors among American surgeons. Ann Surg 2010;251:995–1000 19934755 10.1097/SLA.0b013e3181bfdab3

[21] Chong RIH, Yaow CYL, Chong NZY, Yap NLX, Hong ASY, Ng QX et al Scoping review of the second victim syndrome among surgeons: understanding the impact, responses, and support systems. Am J Surg 2024;229:5–14 37838505 10.1016/j.amjsurg.2023.09.045

[22] Endler NS, Parker JDA. Assessment of multidimensional coping: task, emotion, and avoidance strategies. Psychol Assess 1994;6:50–60

[23] Busch IM, Moretti F, Purgato M, Barbui C, Wu AW, Rimondini M. Dealing with adverse events: a meta-analysis on second victims’ coping strategies. J Patient Saf 2020;16:e51–e60 32168267 10.1097/PTS.0000000000000661

[24] Scott SD, Hirschinger LE, Cox KR, McCoig M, Brandt J, Hall LW. The natural history of recovery for the healthcare provider “second victim” after adverse patient events. BMJ Qual Saf 2009;18:325–330 10.1136/qshc.2009.032870 19812092

[25] Luu S, Patel P, St-Martin L, Leung AS, Regehr G, Murnaghan ML et al Waking up the next morning: surgeons’ emotional reactions to adverse events. Med Educ 2012;46:1179–1188 23171260 10.1111/medu.12058

[26] Edrees H, Connors C, Paine L, Norvell M, Taylor H, Wu AW. Implementing the RISE second victim support programme at the Johns Hopkins Hospital: a case study. BMJ Open 2016;6:e011708 10.1136/bmjopen-2016-011708 PMC5051469 27694486

[27] Scott SD, Hirschinger LE, Cox KR, McCoig M, Hahn-Cover K, Epperly KM et al Caring for our own: deploying a system wide second victim rapid response team. Jt Comm J Qual Patient Saf 2010;36:233–240 20480757 10.1016/s1553-7250(10)36038-7

[28] Busch IM, Moretti F, Purgato M, Barbui C, Wu AW, Rimondini M. Psychological and psychosomatic symptoms of second victims of adverse events: a systematic review and meta-analysis. J Patient Saf 2020;16:e61–e74 30921046 10.1097/PTS.0000000000000589 PMC7386870

[29] Ong TSK, Goh CN, Tan EKYE, Sivanathan KA, Tang ASP, Tan HK et al Second victim syndrome among healthcare professionals: a systematic review of interventions and outcomes. J Healthc Leadersh 2025;17:225–239 40485771 10.2147/JHL.S526565 PMC12145115

[30] Page MJ, McKenzie JE, Bossuyt PM, Boutron I, Hoffmann TC, Mulrow CD et al The PRISMA 2020 statement: an updated guideline for reporting systematic reviews. BMJ 2021;372:n71 33782057 10.1136/bmj.n71 PMC8005924

[31] Spencer L, Ritchie J, Lewis J, Dillon L. Quality in Qualitative Evaluation: A Framework for Assessing Research Evidence. London: Prime Minister’s Strategy Unit Cabinet Office, 2003

[32] Hong QN, Fàbregues S, Bartlett G, Boardman F, Cargo M, Dagenais P et al The Mixed Methods Appraisal Tool (MMAT) version 2018 for information professionals and researchers. Education for Information 2018;34:285–291

[33] Akyol C, Celik SU, Koc MA, Bayindir DS, Gocer MA, Karakurt B et al The impact of patient deaths on general surgeons’ psychosocial well-being and surgical practices. Front Surg 2022;9:898274 35574543 10.3389/fsurg.2022.898274 PMC9096651

[34] Berman L, Rialon KL, Mueller CM, Ottosen M, Weintraub A, Coakley B et al Supporting recovery after adverse events: an essential component of surgeon well-being. J Pediatr Surg 2021;56:833–838 33454081 10.1016/j.jpedsurg.2020.12.031

[35] Biggs S, Waggett HB, Shabbir J. Impact of surgical complications on the operating surgeon. Colorectal Dis 2020;22:1169–1174 32065472 10.1111/codi.15021

[36] Chauvet P, Figuier C, Lambert C, Bourdel N, Canis M. Surgical complications and their impact for gynecological surgeons. Int J Gynaecol Obstet 2023;160:1001–1006 36087015 10.1002/ijgo.14451

[37] Choi SHJ, Yan TD, Misskey J, Chen JC. The emotional impact and coping mechanisms following adverse patient events among Canadian vascular surgeons and trainees. Vasc Endovascular Surg 2024;58:294–301 37878392 10.1177/15385744231209914

[38] Chung DE, Kaushik D, Kobashi K, Leppert JT, Thavaseelan S. Complications: the experience of the urologic surgeon. Urol Pract 2024;11:606–612 38899663 10.1097/UPJ.0000000000000616

[39] Collings R, Potter C, Gebski V, Janda M, Obermair A. The impact of surgical complications on obstetricians’ and gynecologists’ well-being and coping mechanisms as second victims. Am J Obstet Gynecol 2025;232:104.e1–104.e12 10.1016/j.ajog.2024.07.043 39111518

[40] D’Angelo JD, Lund S, Busch RA, Tevis S, Mathis KL, Kelley SR et al Coping with errors in the operating room: intraoperative strategies, postoperative strategies, and sex differences. Surgery 2021;170:440–445 33810853 10.1016/j.surg.2021.02.035

[41] Drudi LM, D’Oria M, Bath J, Nispen JV, Smeds MR. Postoperative complications and their association with post-traumatic stress disorder in academic vascular surgeons. J Vasc Surg 2023;77:899–905.e1 36402248 10.1016/j.jvs.2022.10.056

[42] Han K, Bohnen JD, Peponis T, Martinez M, Nandan A, Yeh DD et al The surgeon as the second victim? Results of the Boston intraoperative adverse events surgeons’ attitude (BISA) study. J Am Coll Surg 2017;224:1048–1056 28093300 10.1016/j.jamcollsurg.2016.12.039

[43] He H, Lin C, Li R, Zang L, Huang X, Liu F. Surgeons’ mental distress and risks after severe complications following radical gastrectomy in China: a nationwide cross-sectional questionnaire. Int J Surg 2023;109:2179–2184 37158145 10.1097/JS9.0000000000000463 PMC10442099

[44] Jain G, Sharma D, Agarwal P, Agrawal V, Yadav SK, Tenzin T et al “Second victim” syndrome among the surgeons from South Asia. Indian J Surg 2022;84:40–46

[45] Khansa I, Pearson GD. Coping and recovery in surgical residents after adverse events: the second victim phenomenon. Plast Reconstr Surg Glob Open 2022;10:e4203 35356044 10.1097/GOX.0000000000004203 PMC8939915

[46] Lin JS, Olutoye OO, Samora JB. To err is human, but what happens when surgeons err? J Pediatr Surg 2023;58:496–502 35914964 10.1016/j.jpedsurg.2022.06.019

[47] McLaren O, Perkins C, Alderson D. The effect of surgical complications on ENT trainees. J Laryngol Otol 2021;135:293–296 33769237 10.1017/S0022215121000797

[48] O’Meara S, D’Arcy F, Dowling C, Walsh K. The psychological impact of adverse events on urology trainees. Ir J Med Sci 2022;192:1819–1824 36329289 10.1007/s11845-022-03202-8 PMC9633123

[49] Patel AM, Ingalls NK, Mansour MA, Sherman S, Davis AT, Chung MH. Collateral damage: the effect of patient complications on the surgeon’s psyche. Surgery 2010;148:824–830 20727563 10.1016/j.surg.2010.07.024

[50] Pinto A, Faiz O, Bicknell C, Vincent C. Acute traumatic stress among surgeons after major surgical complications. Am J Surg 2014;208:642–647 25241953 10.1016/j.amjsurg.2014.06.018

[51] Sandhu H, Foote DC, Evans J, Santosa KB, Kemp MT, Donkersloot JN et al “The story I will never forget”: critical incident narratives in surgical residency. Ann Surg 2023;277:e496–e502 34534986 10.1097/SLA.0000000000005219

[52] Sikakulya FK, Muhumuza J, Vivalya BMN, Mambo SB, Kamabu LK, Muteke JK et al Psychosocial impact of surgical complications and the coping mechanisms among surgeons in Uganda and Eastern Democratic Republic of the Congo. PLOS Glob Public Health 2024;4:e0003180 38683841 10.1371/journal.pgph.0003180 PMC11057973

[53] Sligter LM, van Steijn ME, Scheepstra KW, Dijksman LM, Koot HW, van Pampus MG. Mental-health, coping and support following adverse events on the work-floor: a cross-sectional study among Dutch orthopaedic surgeons. Acta Orthop Belg 2020;86:349–362 33581017

[54] Thompson CV, Naumann DN, Fellows JL, Bowley DM, Suggett N. Post-traumatic stress disorder amongst surgical trainees: an unrecognised risk? Surgeon 2017;15:123–130 26482084 10.1016/j.surge.2015.09.002

[55] Turner K, Bolderston H, Thomas K, Greville-Harris M, Withers C, McDougall S. Impact of adverse events on surgeons. Br J Surg 2022;109:308–310 35084452 10.1093/bjs/znab447

[56] Varughese E, Janda M, Obermair A. Can the use of quality assurance tools reduce the impact of surgical complications on the well-being of obstetricians and gynaecologists in Australia and New Zealand? Aust N Z J Obstet Gynaecol 2014;54:30–35 24359293 10.1111/ajo.12162

[57] Yaow CYL, Ng QX, Chong RIH, Ong C, Chong NZY, Yap NLX et al Intraoperative adverse events among surgeons in Singapore: a multicentre cross-sectional study on impact and support. BMC Health Serv Res 2024;24:512 38659030 10.1186/s12913-024-10998-x PMC11040834

[58] Al-Ghunaim T, Johnson J, Biyani CS, O’Connor DB. Burnout in surgeons: a qualitative investigation into contributors and potential solutions. Int J Surg 2022;101:106613 35421612 10.1016/j.ijsu.2022.106613

[59] Balogun JA, Bramall AN, Bernstein M. How surgical trainees handle catastrophic errors: a qualitative study. J Surg Educ 2015;72:1179–1184 26073715 10.1016/j.jsurg.2015.05.003

[60] Balogun JA, Adekanmbi AA, Balogun FM. Surgical residents as “second victims” following exposure to medical errors in a tertiary health training facility in Nigeria: a phenomenology study. Patient Saf Surg 2023;17:18 37464356 10.1186/s13037-023-00370-z PMC10353142

[61] Bamdad MC, Vitous CA, Rivard SJ, Anderson M, Lussiez A, De Roo A et al What we talk about when we talk about coping: a qualitative study of surgery residents’ coping following complications and deaths. Ann Surg 2023;278:e422–e428 36994739 10.1097/SLA.0000000000005854 PMC10363203

[62] Lu PW, Columbus AB, Fields AC, Melnitchouk N, Cho NL. Gender differences in surgeon burnout and barriers to career satisfaction: a qualitative exploration. J Surg Res 2020;247:28–33 31810639 10.1016/j.jss.2019.10.045

[63] Øyri SF, Søreide K, Søreide E, Tjomsland O. Learning from experience: a qualitative study of surgeons’ perspectives on reporting and dealing with serious adverse events. BMJ Open Qual 2023;12, e002368 10.1136/bmjoq-2023-002368 PMC10254780 37286299

[64] Hsiao L-H, Kopar PK. Surgeon perception and attitude towards the moral imperative of institutionally addressing second victim syndrome in surgery. J Am Coll Surg 2025;240:221–228 39133012 10.1097/XCS.0000000000001191

[65] Ginzberg SP, Gasior JA, Passman JE, Stein J, Keddem S, Soegaard Ballester JM et al Surgeon and surgical trainee experiences after adverse patient events. JAMA Netw Open 2024;7:e2414329 38829617 10.1001/jamanetworkopen.2024.14329 PMC11148685

[66] Vitous CA, Byrnes ME, De Roo A, Jafri SM, Suwanabol PA. Exploring emotional responses after postoperative complications: a qualitative study of practicing surgeons. Ann Surg 2022;275:e124–e131 33443904 10.1097/SLA.0000000000004041 PMC9437841

[67] Pinto A, Faiz O, Bicknell C, Vincent C. Surgical complications and their implications for surgeons’ well-being. Br J Surg 2013;100:1748–1755 24227360 10.1002/bjs.9308

[68] Egbe A, El Boghdady M. Anxiety and depression in surgeons: a systematic review. Surgeon 2024;22:6–17 37852902 10.1016/j.surge.2023.09.009

[69] Fall F, Hu YY, Walker S, Baertschiger R, Gaffar I, Saltzman D et al Peer support to promote surgeon well-being: the APSA program experience. J Pediatr Surg 2024;59:1665–1671 38272766 10.1016/j.jpedsurg.2023.12.022

[70] El Hechi MW, Bohnen JD, Westfal M, Han K, Cauley C, Wright C et al Design and impact of a novel surgery-specific second victim peer support program. J Am Coll Surg 2020;230:926–933 31857209 10.1016/j.jamcollsurg.2019.10.015

[71] Bisset CN, Ferguson E, MacDermid E, Stein SL, Yassin N, Dames N et al Exploring variation in surgical practice: does surgeon personality influence anastomotic decision-making? Br J Surg 2022;109:1156–1163 35851801 10.1093/bjs/znac200 PMC10364753

[72] Bisset CN, Moug SJ, Oliphant R, Dames N, Cleland J. Surgeon perceptions of personality as an influencing factor on anastomotic decision-making: a qualitative analysis. Colorectal Dis 2024;26:1608–1616 39162024 10.1111/codi.17078

[73] Bisset CN, Moug SJ, Oliphant R, Dames N, Parson S, Cleland J. Influencing factors in surgical decision-making: a qualitative analysis of colorectal surgeons’ experiences of postoperative complications. Colorectal Dis 2024;26:987–993 38485203 10.1111/codi.16943

[74] Yule S, Flin R, Paterson-Brown S, Maran N. Non-technical skills for surgeons in the operating room: a review of the literature. Surgery 2006;139:140–149. 16455321 10.1016/j.surg.2005.06.017

[75] Luton OW, James OP, Mellor K, Eley C, Hopkins L, Robinson DBT et al Enhanced stress-resilience training for surgical trainees. BJS Open 2021;5:zrab054 34323917 10.1093/bjsopen/zrab054 PMC8320339

[76] Lebares CC, Coaston TN, Delucchi KL, Guvva EV, Shen WT, Staffaroni AM et al Enhanced stress resilience training in surgeons: iterative adaptation and biopsychosocial effects in 2 small randomized trials. Ann Surg 2021;273:424–432 32773637 10.1097/SLA.0000000000004145 PMC7863698

[77] Ozer EJ, Best SR, Lipsey TL, Weiss DS. Predictors of posttraumatic stress disorder and symptoms in adults: a meta-analysis. Psychol Bull 2003;129:52–73. 12555794 10.1037/0033-2909.129.1.52

[78] DiGangi JA, Gomez D, Mendoza L, Jason LA, Keys CB, Koenen KC. Pretrauma risk factors for posttraumatic stress disorder: a systematic review of the literature. Clin Psychol Rev 2013;33:728–744. 23792469 10.1016/j.cpr.2013.05.002

[79] Orri M, Revah-Lévy A, Farges O. Surgeons’ emotional experience of their everyday practice—a qualitative study. PLoS One 2015;10:e0143763 26600126 10.1371/journal.pone.0143763 PMC4657990

[80] Shanafelt TD, Kaups KL, Nelson H, Satele DV, Sloan JA, Oreskovich MR et al An interactive individualized intervention to promote behavioral change to increase personal well-being in US surgeons. Ann Surg 2014;259:82–88 23979287 10.1097/SLA.0b013e3182a58fa4 PMC4333681

[81] Hodson N . Reflective practice and gross negligence manslaughter. Br J Gen Pract 2019;69:135 30819743 10.3399/bjgp19X701561 PMC6400605

[82] Jackson TN, Morgan JP, Jackson DL, Cook TR, McLean K, Agrawal V et al The crossroads of posttraumatic stress disorder and physician burnout: a national review of United States trauma and nontrauma surgeons. Am Surg 2019;85:127–135 30819287

[83] Menon NK, Shanafelt TD, Sinsky CA, Linzer M, Carlasare L, Brady KJS et al Association of physician burnout with suicidal ideation and medical errors. JAMA Netw Open 2020;3:e2028780 33295977 10.1001/jamanetworkopen.2020.28780 PMC7726631

