# Supplementary material for: Second victim syndrome in surgeons: systematic review and meta-analysis of the impact of adverse events on surgeons
Source: Br J Surg. 2026 Jan 7;113(1):znaf258. doi: 10.1093/bjs/znaf258 (PMC12777971; doi:10.1093/bjs/znaf258)
Supplement: znaf258_Supplementary_Data [file znaf258_supplementary_data.docx]

# Second Victim Syndrome in Surgeons: a systematic review and meta-analysis of the impact of adverse clinical events on surgeons

# J Bryan^1,2^, A Ketley^1^, K Cavanagh^3^, C Bisset^4^, S Moug^4,5^, L Wyld^1,2^, J Morgan^1,2^

1. The University of Sheffield Faculty of Medicine Dentistry and Health, School of Medicine and Population Health,

Sheffield

2. Doncaster and Bassetlaw Teaching Hospitals NHS Foundation Trust

3. School of Psychology, University of Sussex, Brighton

4. University of Glasgow, School of Medicine, Dentistry & Nursing

5. Royal Alexandra Hospital, Paisley, NHS Scotland

**Corresponding author.** James Bryan **ORCID ID:** 0000-0002-7979-6501

**Supplementary Materials - Index**

| **Supplementary Methods** |  |
| --- | --- |
| Search Strategy | *pag. 2,3* |
| **Supplementary Figures and Tables** |  |
| **Supplementary table 1:** Summary of risk of bias assessment using MMAT tool | *pag. 4* |
| **Supplementary table 2:** Representative quotes on the impact of adverse events on the surgeon | *pag. 5-7* |
| **Supplementary table 3:** Representative quotes of coping strategies employed by surgeons | *pag. 8-10* |
| **Supplementary table 4:** Representative quotes of barriers affecting engagement with support strategies | *pag. 11* |
| **References** | *Pag. 12* |
|  |  |

**Supplementary Methods**

**Search strategy**

Search

(( "second victim" OR "psychological impact" OR "psychological stress" OR "burnout" OR "compassion fatigue" OR "post-traumatic stress" OR PTSD OR "coping mechanisms" OR resilience)

AND

(surgeon OR "surg* registrar" OR "surg* resident" OR "surg* trainee")

AND

("adverse event*" OR complication* OR "medical error*" OR "patient harm" OR "operative error*" OR "unexpected outcome*"))

1. PUBMED:

(("second victim"[tiab] OR "psychological impact"[tiab] OR "psychological stress"[MeSH] OR "burnout"[MeSH] OR "compassion fatigue"[tiab] OR "post-traumatic stress"[tiab] OR "PTSD"[tiab] OR "coping mechanisms"[tiab] OR "resilience"[tiab]) AND (surgeon[tiab] OR "surg* registrar"[tiab] OR "surg* resident"[tiab] OR "surg* trainee"[tiab]) AND ("adverse event*"[tiab] OR complication*[tiab] OR "medical error*"[MeSH] OR "patient harm"[tiab] OR "operative error*"[tiab] OR "unexpected outcome*"[tiab]))

2. Cochrane Library:

("second victim" OR "psychological impact" OR "psychological stress" OR "burnout" OR "compassion fatigue" OR

"post-traumatic stress" OR PTSD OR "coping mechanisms" OR resilience)

AND

(surgeon OR "surg* registrar" OR "surg* resident" OR "surg* trainee")

AND

("adverse event*" OR complication* OR "medical error*" OR "patient harm" OR "operative error*" OR "unexpected outcome*")

3. APA PsycInfo via OVID:

("second victim" OR "psychological impact" OR "psychological stress" OR "burnout" OR "compassion fatigue" OR "post-traumatic stress" OR PTSD OR "coping mechanisms" OR resilience) AND (surgeon OR "surg* registrar" OR "surg* resident" OR "surg* trainee") AND ("adverse event*" OR complication* OR "medical error*" OR "patient harm" OR "operative error*" OR "unexpected outcome*"))

4. Web of Science:

TS=("second victim" OR "psychological impact" OR "psychological stress" OR "burnout" OR "compassion fatigue" OR

"post-traumatic stress" OR PTSD OR "coping mechanisms" OR resilience)

AND

TS=(surgeon OR "surg* registrar" OR "surg* resident" OR "surg* trainee")

AND

TS=("adverse event*" OR complication* OR "medical error*" OR "patient harm" OR "operative error*" OR "unexpected outcome*")

5. SCOPUS

(( "second victim" OR "impact" OR "psychological stress" OR "burnout" OR "compassion fatigue" OR "post-traumatic stress" OR PTSD OR "coping mechanisms" OR resilience)

AND

(surgeon OR "surg* registrar" OR "surg* resident" OR "surg* trainee")

AND

("adverse event*" OR complication* OR "medical error*" OR "patient harm" OR "operative error*" OR "unexpected outcome*"))

**Supplementary Figures and Tables**

**Supplementary table 1:** Summary of risk of bias assessment using MMAT tool

| **Author, Year** | **Type** | S1 | S2 | 1.1 | 1.2 | 1.3 | 1.4 | 1.5 | 4.1 | 4.2 | 4.3 | 4.4 | 4.5 | 5.1 | 5.2 | 5.3 | 5.4 | 5.5 |
| --- | --- | --- | --- | --- | --- | --- | --- | --- | --- | --- | --- | --- | --- | --- | --- | --- | --- | --- |
| Akyol et al 2022 | Survey |  |  |  |  |  |  |  |  |  |  |  |  |  |  |  |  |  |
| Al-Ghunaim et al 2022 | Semi-Structured interviews |  |  |  |  |  |  |  |  |  |  |  |  |  |  |  |  |  |
| Balogun et al 2015 | Semi-Structured interviews |  |  |  |  |  |  |  |  |  |  |  |  |  |  |  |  |  |
| Balogun et al 2023 | Semi-Structured interviews |  |  |  |  |  |  |  |  |  |  |  |  |  |  |  |  |  |
| Bamdad et al 2023 | Semi-Structured interviews |  |  |  |  |  |  |  |  |  |  |  |  |  |  |  |  |  |
| Berman et al 2021 | Survey |  |  |  |  |  |  |  |  |  |  |  |  |  |  |  |  |  |
| Biggs et al 2020 | Survey |  |  |  |  |  |  |  |  |  |  |  |  |  |  |  |  |  |
| Chauvet et al 2023 | Survey |  |  |  |  |  |  |  |  |  |  |  |  |  |  |  |  |  |
| Choi et al 2024 | Survey |  |  |  |  |  |  |  |  |  |  |  |  |  |  |  |  |  |
| Chung et al 2024 | Survey |  |  |  |  |  |  |  |  |  |  |  |  |  |  |  |  |  |
| Collings et al 2024 | Survey |  |  |  |  |  |  |  |  |  |  |  |  |  |  |  |  |  |
| D’Angelo et al 2021 | Survey |  |  |  |  |  |  |  |  |  |  |  |  |  |  |  |  |  |
| Drudi et al 2023 | Survey |  |  |  |  |  |  |  |  |  |  |  |  |  |  |  |  |  |
| Ginzberg et al 2024 | Survey, Interviews |  |  |  |  |  |  |  |  |  |  |  |  |  |  |  |  |  |
| Han et al 2017 | Survey |  |  |  |  |  |  |  |  |  |  |  |  |  |  |  |  |  |
| He et al 2023 | Survey |  |  |  |  |  |  |  |  |  |  |  |  |  |  |  |  |  |
| Hsiao et 2024 | survey , focus group and semi-structured interviews |  |  |  |  |  |  |  |  |  |  |  |  |  |  |  |  |  |
| Jain et al | Survey |  |  |  |  |  |  |  |  |  |  |  |  |  |  |  |  |  |
| Khansa et al 2022 | Survey |  |  |  |  |  |  |  |  |  |  |  |  |  |  |  |  |  |
| Lin et al 2023 | Survey |  |  |  |  |  |  |  |  |  |  |  |  |  |  |  |  |  |
| Lu et al 2020 | Semi-structured interviews |  |  |  |  |  |  |  |  |  |  |  |  |  |  |  |  |  |
| Luu et al 2012 | Semi-structured interviews |  |  |  |  |  |  |  |  |  |  |  |  |  |  |  |  |  |
| McLaren et al 2021 | Survey |  |  |  |  |  |  |  |  |  |  |  |  |  |  |  |  |  |
| O’Meara et al 2023 | Survey |  |  |  |  |  |  |  |  |  |  |  |  |  |  |  |  |  |
| Oyri et al 2023 | Semi-structured interviews |  |  |  |  |  |  |  |  |  |  |  |  |  |  |  |  |  |
| Patel et al 2010 | Survey |  |  |  |  |  |  |  |  |  |  |  |  |  |  |  |  |  |
| Pinto et al 2013 | Semi-structured interviews |  |  |  |  |  |  |  |  |  |  |  |  |  |  |  |  |  |
| Pinto et al 2014 | Survey |  |  |  |  |  |  |  |  |  |  |  |  |  |  |  |  |  |
| Sandhu et al 2023 | Survey |  |  |  |  |  |  |  |  |  |  |  |  |  |  |  |  |  |
| Sikakulya et al 2024 | Survey |  |  |  |  |  |  |  |  |  |  |  |  |  |  |  |  |  |
| Sligter et al 2020 | Survey |  |  |  |  |  |  |  |  |  |  |  |  |  |  |  |  |  |
| Thomson et al 2017 | Survey |  |  |  |  |  |  |  |  |  |  |  |  |  |  |  |  |  |
| Turner et al 2022 | Survey |  |  |  |  |  |  |  |  |  |  |  |  |  |  |  |  |  |
| Varughese et al 2023 | Survey |  |  |  |  |  |  |  |  |  |  |  |  |  |  |  |  |  |
| Vitous et al 2022 | Semi-Structured interviews |  |  |  |  |  |  |  |  |  |  |  |  |  |  |  |  |  |
| Yaow et al 2024 | Survey |  |  |  |  |  |  |  |  |  |  |  |  |  |  |  |  |  |
|  |  |  |  |  |  |  |  |  |  |  |  |  |  |  |  |  |  |  |
| **Key** | Yes |  |  |  |  |  |  |  |  |  |  |  |  |  |  |  |  |  |
|  | No |  |  |  |  |  |  |  |  |  |  |  |  |  |  |  |  |  |
|  | Not Sure |  |  |  |  |  |  |  |  |  |  |  |  |  |  |  |  |  |

**Supplementary table 2:** Representative quotes on the impact of adverse events on the surgeon

| **Theme** |  | **Quote** |
| --- | --- | --- |
| Psychosomatic | Sleep disturbance | ‘[I feel] a pall over everything, like I couldn’t sleep without thinking about it... I grieve for how badly it makes me feel. I’m always saying I’ve got to get out of this business because it’s hard. It’s depressing...’[(1)](https://www.zotero.org/google-docs/?WqZ6YK)  “Yeah, always feel awful. Yeah, it’s easily the worst part of my job. And I think most people would say that. And I think that a lot of people don’t understand how it affects physicians. And we oftentimes internalize it. But it, although it sounds cliché, it really does keep, at least keep me up at night. And I know it keeps other people up at night and lose sleep over complications.” [(2)](https://www.zotero.org/google-docs/?FGpDsK)  “I had a hard time sleeping for a while after a complication. Sometimes I would feel my heart racing during the day or it would be difficult to breathe.” [(3)](https://www.zotero.org/google-docs/?nPJdGR)  “It’s one of those things where you wake up in the middle of night, you’re like, checklist. Could I have done this? Could I have done this? Could I have done this?” [(4)](https://www.zotero.org/google-docs/?Dzkfym) |
| Psychological | Anxiety | ‘There’s tachycardia. There’s some tachycardia and some unease. ...there’s still that anxiety... It’s the same sort of feeling you get if something tragic happens in your life, somebody important in your life is no longer there. It’s a different feeling but it’s in the same category of things. That’s when I sort of feel that I need to run and hide.’[(1)](https://www.zotero.org/google-docs/?jfcpkk) |
|  | Rumination | ‘I honestly think I almost crashed into four parked cars before I got out of the parking garage that day. I was so distraught...’[(1)](https://www.zotero.org/google-docs/?WLVEAw)  ‘Did I goof something or did I miss something? Is it a technical problem? So I relive the operation and I go through the critical parts of the operation.’ [(1)](https://www.zotero.org/google-docs/?Aht92f)  ‘Until the pall had lifted and [the patient] sorted, I couldn’t think about anything else.’[(1)](https://www.zotero.org/google-docs/?umh9u7)  ‘So I do it just to punish myself, just to torture myself, just to flagellate myself. I go over and over and I beat myself up. And I tell myself I’m not worthy and I’m a piece of shit and I’m crap. And I should have been a truck driver instead of a [specialty] surgeon.’ [(1)](https://www.zotero.org/google-docs/?JpsiaK)  ‘I'm trying to help my kids with homework and I'm thinking about my patient and I'm trying to sit and have dinner with them and hear about their day and I'm thinking about my patient’[(4)](https://www.zotero.org/google-docs/?VVp6aM) |
|  | Low self-esteem | ‘It’s like I failed. I got a C. I got a D... I’m not entitled to wear my lab coat and my scrubs and be a surgeon... You just feel personally devalued.’[(1)](https://www.zotero.org/google-docs/?a9Mg5n)  ‘It’s not so much anymore that I worry am I a bad surgeon, are people thinking I’m a bad surgeon.’[(1)](https://www.zotero.org/google-docs/?Y5neVZ)  “I’m pretty analytical, and so I think my first reaction is to kind of emotionally, to the best of my ability, emotionally withdraw myself and try and figure out why something happened. And sometimes I’m able to figure out that this happened because of something that wasn’t my fault. And then I feel bad for the patient, but I don’t really internalize that guilt. But then sometimes I figure out that it’s something that I’ve did, done, and that weighs on me. Yeah, it makes me less confident.”[(2)](https://www.zotero.org/google-docs/?geIL0k) |
|  | Fear (Professional reputation/medico-legal repercussions | “ think there was a lot of fear. And, I was at a point where I was junior. So, I didn’t quite understand that complications happen no matter what you do. And, a complication did happen. I didn’t really know how to deal with it, at that point in my career"[(5)](https://www.zotero.org/google-docs/?jHpWDx)  “...quite often you think what have I done wrong, am I in trouble for this ...is this an error that I’ve made that’s unforgivable and is it going to affect people’s professional opinion of me …”[(6)](https://www.zotero.org/google-docs/?1CoHZi)  “And then there, you can be sued. You can actually be sued. So,there’s all these things which, you know, interface. And obviously, the worst thing is to be sued and be told that you ruined somebody’s life, and you probably did.” [(2)](https://www.zotero.org/google-docs/?IpxGFZ)  “And then a little bit of it comes into also like anxiety of, okay, you know, are they going to potentially have some retaliation for this, and also on top of it is your reputation as a surgeon in the community, are people going to say, oh, he’s kind of, reckless as a surgeon because he had a complication?”[(2)](https://www.zotero.org/google-docs/?MQPuCF) |
|  | Sadness | “You feel depressed In cases of a patient dying, you’re saddened by it. It’s kind of the back of your mind for days, for weeks,sometimes longer.It takes a very deep emotional toll.”[(2)](https://www.zotero.org/google-docs/?qNM0kB)  “I was not myself, I just felt that if anything should happen to this child, I was the cause of the patient’s death but luckily nothing happened...I gradually gained back my confidence.“ [(7)](https://www.zotero.org/google-docs/?x2tTOP)  “I was unhappy...unhappiness is a stressful situation...”[(7)](https://www.zotero.org/google-docs/?DyTvxV) |
|  | Burnout | “The experience was terrible...it takes a lot of courage to keep practicing with those experiences because it can be demoralizing...”[(7)](https://www.zotero.org/google-docs/?Xgu9Kw)  “Complications is still what makes this job most difficult. Makes you think every once in a while, geez, I should have gone into... radiology or pathology.”[(2)](https://www.zotero.org/google-docs/?MySnKS)  “I think that it does take a mass of emotional pull, I’ll be honest, and it is difficult, and I had a pretty big period of burnout the last couple of months, and I actually had to adjust my schedule... in order to compensate for that because I just wasn’t rebounding like I had in the past. So, I had to take some more time off.”[(2)](https://www.zotero.org/google-docs/?z8WQDz)  “You know, I’m thinking as you get older as a surgeon, because I’m older and, you know, nearing retirement as, should I be operating this? You know, so you start to think that. Am I getting too old? Am I having a complication because I’m just not at the top of my game anymore? So those sorts of things come in too at the end of your career as well.” [(2)](https://www.zotero.org/google-docs/?tkj3TW) |
| Professional | Change in practice (technical) | “To be more careful and know my limit... also do a proper pre op evaluation...know when to call your superiors for help.“ [(7)](https://www.zotero.org/google-docs/?QK4Jfl)  “It made me to be more detailed and more careful, in terms of preparing a patient for surgery...”[(7)](https://www.zotero.org/google-docs/?4EnwXD)  ...I have decided to have a protocol written for each of the cases I will be handling later in life... the same scrutiny that applies to one patient will be applied to others to minimize each of these errors in the future.“ [(7)](https://www.zotero.org/google-docs/?MLTgN0)  “Every major complication I’ve had in residency has in some way changed my practice, and maybe that’s not a good thing. Maybe that’s kind of reactionary. Some of them are way bigger changes than others. Some just slightly move the needle and some like are huge adjustments. I think that like kind of the process they go through to like deal with complications.” [(8)](https://www.zotero.org/google-docs/?3gq56m)  ‘I’ve written a lot of SOPs [standard operating procedures]. In my OR [operating room] I have them and I will put in if I think maybe this piece wasn’t right. I will put in a line item for that and pay more attention... I’m sort of... how will I deal with that one next time, maybe that’s my coping mechanism... how I can do it better... I have an SOP and I have databases on them.’ [(1)](https://www.zotero.org/google-docs/?SkDGVc)  “ ...Well it might make me much less prone to taking any form of risk ...and sometimes that’s not necessarily in the best interest of the patient ...and I see this behaviour in my colleagues at a consultant level and down through to the registrars and even the SHOs [senior house officers] …”[(6)](https://www.zotero.org/google-docs/?gcMLu2)  Every time a complication develops it just makes you think more and plan more for next time. It just means more reflection. So whenever I face a complication or unexpected outcome, I try to reflect more on the cases to see what I could have done differently.[(7)](https://www.zotero.org/google-docs/?juHPUb) |
|  | Change in practice  (non-technical) | “It’s made me more open to talking with patients, certainly about risks but also if something happens just talking to them with, about that at bedside. So like, and not standing over them but pulling up a chair and saying, here’s what either happened or I think is going on. Here’s what we’re going to do to get you through this, and, you know, again, here’s our action plan and then listening. So I feel like those are the skills that have really kind of blossomed from dealing with these problems. These I know that certainly will help them, but it also helps me there as well.”[(8)](https://www.zotero.org/google-docs/?fgIiVg)  ‘I think that you really grow as a person when you think about... when you see somebody suffering... It makes me I think more appreciative of the tremendous distress it causes and I think that I react to that. I don’t try to run away and hide from it. I think I engage it and try to make things better for the patients.’[(1)](https://www.zotero.org/google-docs/?9RzEdZ)  It has affected me a lot, I think I have learnt to listen to even the least person in the team.[(7)](https://www.zotero.org/google-docs/?Q8DpF6) |

**Supplementary table 3: Representative quotes of coping strategies employed by surgeons**

| **Theme** |  | **Quote** |
| --- | --- | --- |
| Internal | Inevitability | “I guess I felt a little better because I have a mind-set that I can make mistakes if probably I expect myself to be perfect, I will feel a lot worse...” [(7)](https://www.zotero.org/google-docs/?HmpLqP)  “Even the best anastomosis can leak, right?... so you’re trying to do as good as you can, but there’s always a possibility of complication” [(8)](https://www.zotero.org/google-docs/?h7aXQg)  “You can do the best that you can every single day, and you’re still going to have people that don’t do well, whether it’s the system or their disease process, or something that you did, not maliciously, but you know, because you’re a human being and you’re fallible.”[(8)](https://www.zotero.org/google-docs/?M66IBN)  “I better recognize now that there’s only so much that you can control and that there’s a certain component of all surgeries that, regardless of how much you try, just can’t control all the variables, necessarily always have that good outcome...maybe now I’m a little bit more accepting that it’s not always a personal mistake or not always a true technical problem that led to the complication, that there’s a whole host of things that you can’t necessarily make perfect, and that complications are a normal part of doing big surgeries on people, especially people who aren’t healthy to begin with.”[(8)](https://www.zotero.org/google-docs/?DK7wGX)  As a senior resident, I’ve kind of come to terms with the fact that like bad things happen. You can do your best and be a good surgeon, and bad things will still happen. You can do your best and be a good surgeon and still make mistakes sometimes.” [(8)](https://www.zotero.org/google-docs/?C2il3F) |
|  | Contextualisation | “I actually have saved some files of screenshots of very nice reviews that patients—as much as I hate that I’m being rated like a restaurant. I have saved some of them that are meaningful and kind. I will reread those to try and find a perspective.” (63)  ‘If they had a PE [pulmonary embolus] and we’d had them on prophylaxis, there’s a big difference there. If someone had a PE and we hadn’t had them on prophylaxis, that would make me feel terrible.’ [(1)](https://www.zotero.org/google-docs/?3qRaR9)  ‘ ...what do most surgeons like about surgery? It’s the technical aspect of it ...And if by your own hand, own error and judgement someone has suffered, then of course you’re going to bear that complication as a greater burden than say there was some unexpected anatomy ... putting in a stitch badly that tears a vein, you’re going to kick yourself for that a bit more ...’[(6)](https://www.zotero.org/google-docs/?izTDPZ) |
|  | Avoidance/ Compartmentalisation | “Keep work at work and keep home at home” [(8)](https://www.zotero.org/google-docs/?VTjCkx)  “I think in, it could make you really sad over time and really kind of burned-out. And it can also make you stop caring, just become desensitized to it, which also isn’t good.”[(8)](https://www.zotero.org/google-docs/?8P0YaI)  “I’m pretty good at compartmentalizing, and so I think, you know, that has helped me personally just be able to continue with other things in my life without letting it affect me too much.” [(8)](https://www.zotero.org/google-docs/?c5P6Vy)  “The other way is really on my weekends off, I just love to be quiet and just have some time to internally process. And I guess the other thing I would say is, I mean, you know, just a hobby like, in my case, I guess, either picking up my guitar or going out for a really nice meal, having just one drink and sitting down and enjoying that meal. You know, just something to kind of divert energy or quiet time to think through that situation. Those are probably the things I do.”[(8)](https://www.zotero.org/google-docs/?Ss79JX) |
|  | Emotional Resilience | “Resiliency for me can just be related to being comfortable with always having to fight the battle, never having it be won, and each day still trying to figure out how to fight it a little bit better than the day before” [(8)](https://www.zotero.org/google-docs/?qnaWQc)  “It was really kind of hard to come back from that, although I, you know, essentially did the next day and did the same procedure multiple times again. And I think that’s kind of what you have to do as surgeons, but also I think it was hard for me to kind of, you know, maintain your sense of self-confidence when you have an outcome like that.” [(8)](https://www.zotero.org/google-docs/?H8mw2p)  I think in general people who go into medicine, we’re thought of and we think of ourselves as these strong people who are hard workers and can get through things, and that’s why we’ve, we’re in the profession that we are. And the thing is, is that, you know, we’re not superheroes, and there’s only so much that one person can take in and deal with. So I think that’s just, it’s part of the pride of, you know, doctors in general.” [(8)](https://www.zotero.org/google-docs/?rOovM7)  “I think we’re generally poor at estimating the cumulative impact that these stressful events have on life... Surgeons pride themselves on being able to tough things out and continue to function.”[(1)](https://www.zotero.org/google-docs/?6Wdkqj)  “Sure, of course, you know, I do feel bad when complications happen, and I have to be very open and honest, and I always tell both the patient’s family and the patient that, you know, well, this was an undesired outcome, you know, this is what happened, and we have to move forward and not move back. But I certainly, I don’t let it hold me down because I can’t let it distract with the next decision that I have to make 10 minutes later. This is when my wife tells me that I have no emotions because I have to keep moving forward. So it doesn’t usually slow me down too much, although I do feel bad.” [(2)](https://www.zotero.org/google-docs/?JNZ5MK) |
| External | Patient and their family | “They can forgive you or at least make you feel like you’re still a good doctor at the end of it” [(8)](https://www.zotero.org/google-docs/?d1mr3n)  So I, the thing that I think makes me feel the best is when I actually just talk with the patient and their family about these things.” [(8)](https://www.zotero.org/google-docs/?GtOHCF)  “It is very hard at that moment to say that the surgical procedure did not go well but one must be honest and explain what happened and say that I am deeply sorry.”[(9)](https://www.zotero.org/google-docs/?fgyyHi)  “I strongly recommend being open with the family because it lifts the weight from your shoulders and will liberate you from hiding something.”[(9)](https://www.zotero.org/google-docs/?IR73lV)  “I talked with the patient and fully admitted that this was not up to standard, and she tackled this much better than I had expected.”[(9)](https://www.zotero.org/google-docs/?xBYACI) |
|  | Systemic/Organisational | “The systemic support ideally should be the normal morbidity/mortality meeting... where someone should own up to those errors, you are appropriately guided...” [(7)](https://www.zotero.org/google-docs/?3KpqFn)  “I believe that bringing too much emotional reactions into a work environment may seem wrong. A culture of openness in an environment must have a more rational basis. Therefore, the complication meetings were established, because they provide a different context.”[(9)](https://www.zotero.org/google-docs/?Tzv3tt) |
|  | External professional | “And so honestly, over the last year I’ve started seeing a counselor. And I go in, I talk about all the crap that’s happened at work, and I feel lots better.” [(8)](https://www.zotero.org/google-docs/?D8rn6O) |
|  | Friends and family | “My husband is a great support, and we often talk about what’s going on at my job, but he’s not in medicine, so there’s only so much true empathizing.” [(4)](https://www.zotero.org/google-docs/?C6wLPg) |
|  | Mentor | In the unit, we do sometimes go over things that happen...when a senior resident tells you that you did the best, it helps to assuage guilt feelings...” [(7)](https://www.zotero.org/google-docs/?Hk0teH)  “I would imagine there would be a mentor- mentee relationship which will be the best kind of support because it is only a colleague, or a senior colleague that will offer you succor...somebody knowing and accepting that you are a trainee...” [(7)](https://www.zotero.org/google-docs/?iSr17x)  ‘ ...We have a team of three, we do a ward round together and we bounce opinions off each other ... We will happily discuss complex cases and complications of cases. And that’s a very good stress reliever, especially if you’re relatively junior and there’s a very senior person who’s probably seen it all before, that’s very reassuring ...’[(6)](https://www.zotero.org/google-docs/?ra6dRv)  ‘ ...it’s very good to have someone a little more senior that if you have a problem you can say, ‘‘What am I going to do?’’ or ‘‘What happens next?’’ That’s very, very unofficial and it would be nice if it could be made more structured in the Health Service ...’[(6)](https://www.zotero.org/google-docs/?du8iwC) |
|  | Peer | “My colleagues were very supportive, they understood all that happened, they told me the plain truth. They also empathized...I appreciate their role in supporting and correcting me the way they did. [(7)](https://www.zotero.org/google-docs/?aU9AoU)  “It’s probably finding a sounding board, finding colleagues who you can reflect with, talk to about this. And I think that’s one of the most powerful ways to deal with it. Talk to people who can relate to what you’re going through and say I’ve, that’s happened to me too, right. So then you don’t feel alone that you’re the only person that messed up. And I think that’s one of the ways I handle it.” [(8)](https://www.zotero.org/google-docs/?kpbJiq)  “At least my experience was there’s nothing formal about it, but the support came from the relationships that I had with residents and faculty. You know, I work with these people every day, and we’re close with each other. So definitely the faculty, in that case, you know, sat down and we talked about it quite a bit afterwards.” [(8)](https://www.zotero.org/google-docs/?0QXBHF) |

**Supplementary table 4: Representative quotes of barriers affecting engagement with support strategies**

| **Theme** | **Quote** |
| --- | --- |
| Awareness | “Is there support within our department or division? I don’t know. That’s a great question. No one ever talks about it.” [(4)](https://www.zotero.org/google-docs/?mzYy6r) |
| Skepticism | “I have never utilized it. I don’t even know if a resource like that exists. I’m not sure many would use it.”[(4)](https://www.zotero.org/google-docs/?vXJ7t8) |
| Culture | “morbidity and mortality meetings are supposed to be a forum where you can have an open discussion but if anyone believes that they’re only kidding themselves; everybody in that room is very defensive and aggressively pursues an angle that puts them in the best possible light and professional rivalries exist ...I don’t find them cathartic forums for saying that was just terrible wasn’t it” [(6)](https://www.zotero.org/google-docs/?4Kww13)  ‘ ...[hospital managers] march in to the ward and the immediate implication is that you’ve done something wrong. So I don’t believe in blameless culture, that is a total fallacy ...’ [(6)](https://www.zotero.org/google-docs/?dsWBlG)  "To be a surgeon seems to depend on a degree of arrogance and overwhelming self-confidence to allow you to do what we do. And, of course, that's a really double-edged sword’[(5)](https://www.zotero.org/google-docs/?Z09KOn) |
| Stigma | “People don’t talk to each other about these sorts of things. They just—whether it’s complications or even things like, when you’re young attending, the first time you get sued—people just don’t talk about it. It’s like a dirty little secret or something.” [(4)](https://www.zotero.org/google-docs/?86FiUH)  “My network would not be within my department. And perhaps some of that is because I would have concerns about potential retribution or—not retribution, but I don’t want it accounted for. Or, as much as people talk about things being confidential, I would have a fear it wasn’t.” [(4)](https://www.zotero.org/google-docs/?mIfZKa)  If one is afraid of being considered a less good surgeon, or afraid of losing face among colleagues, or not being allowed to operate any more, one would try to avoid talking about it. [(9)](https://www.zotero.org/google-docs/?QW5IME) |
| Unfamiliarity/isolation from colleagues | “I am fortunate to know and trust my colleagues because I had already been a member of the department for 9 years....I could very easily see how that might be difficult if I entered a brand-new space without those established and trusted relationships.”[(4)](https://www.zotero.org/google-docs/?BbYwim)  ‘I know I don’t feel comfortable talking to my colleagues about it. I’m not sure they would be as much support perhaps as they should be.’[(1)](https://www.zotero.org/google-docs/?KoSsxU)  “I feel additional guilt about complications from my senior colleagues.”[(3)](https://www.zotero.org/google-docs/?o30jkT) |

**References**

[1.](https://www.zotero.org/google-docs/?AKJqb5) [Luu S, Patel P, St-Martin L, Leung AS, Regehr G, Murnaghan ML, et al. Waking up the next morning: surgeons’ emotional reactions to adverse events. Medical Education. 2012;46(12):1179–88.](https://www.zotero.org/google-docs/?AKJqb5)

[2.](https://www.zotero.org/google-docs/?AKJqb5) [Vitous CA, Byrnes ME, De Roo A, Jafri SM, Suwanabol PA. Exploring Emotional Responses After Postoperative Complications: A Qualitative Study of Practicing Surgeons. Annals of Surgery. 2022 Jan;275(1):e124.](https://www.zotero.org/google-docs/?AKJqb5)

[3.](https://www.zotero.org/google-docs/?AKJqb5) [Lu PW, Columbus AB, Fields AC, Melnitchouk N, Cho NL. Gender Differences in Surgeon Burnout and Barriers to Career Satisfaction: A Qualitative Exploration. Journal of Surgical Research. 2020 Mar 1;247:28–33.](https://www.zotero.org/google-docs/?AKJqb5)

[4.](https://www.zotero.org/google-docs/?AKJqb5) [Ginzberg SP, Gasior JA, Passman JE, Stein J, Keddem S, Soegaard Ballester JM, et al. Surgeon and Surgical Trainee Experiences After Adverse Patient Events. JAMA Network Open. 2024 June 3;7(6):e2414329.](https://www.zotero.org/google-docs/?AKJqb5)

[5.](https://www.zotero.org/google-docs/?AKJqb5) [Al-Ghunaim T, Johnson J, Biyani CS, O’Connor DB. Burnout in surgeons: A qualitative investigation into contributors and potential solutions. International Journal of Surgery. 2022 May 1;101:106613.](https://www.zotero.org/google-docs/?AKJqb5)

[6.](https://www.zotero.org/google-docs/?AKJqb5) [Pinto A, Faiz O, Bicknell C, Vincent C. Surgical complications and their implications for surgeons’ well-being. British Journal of Surgery. 2013 Dec 1;100(13):1748–55.](https://www.zotero.org/google-docs/?AKJqb5)

[7.](https://www.zotero.org/google-docs/?AKJqb5) [Balogun JA, Adekanmbi AA, Balogun FM. Surgical residents as “second victims” following exposure to medical errors in a tertiary health training facility in Nigeria: a phenomenology study. Patient Saf Surg. 2023 July 18;17:18.](https://www.zotero.org/google-docs/?AKJqb5)

[8.](https://www.zotero.org/google-docs/?AKJqb5) [Bamdad MC, Vitous CA, Rivard SJ, Anderson M, Lussiez A, De Roo A, et al. What We Talk About When We Talk About Coping: A Qualitative Study of Surgery Residents’ Coping following Complications and Deaths. Ann Surg. 2023 Aug 1;278(2):e422–8.](https://www.zotero.org/google-docs/?AKJqb5)

[9.](https://www.zotero.org/google-docs/?AKJqb5) [Øyri SF, Søreide K, Søreide E, Tjomsland O. Learning from experience: a qualitative study of surgeons’ perspectives on reporting and dealing with serious adverse events. BMJ Open Qual [Internet]. 2023 June 7 [cited 2024 Dec 18];12(2). Available from: https://bmjopenquality.bmj.com/content/12/2/e002368](https://www.zotero.org/google-docs/?AKJqb5)
